# Mitochondrial genome evolution in species belonging to the *Phialocephala fortinii* s.l. - *Acephala applanata species* complex

**DOI:** 10.1186/1471-2164-13-166

**Published:** 2012-05-04

**Authors:** Angelo Duò, Rémy Bruggmann, Stefan Zoller, Matthias Bernt, Christoph R Grünig

**Affiliations:** 1 Forest Pathology and Dendrology, Institute of Integrative Biology (IBZ), ETH Zurich, CH-8092, Zurich, Switzerland; 2 Bioinformatics, Department of Biology, University of Berne, Baltzerstrasse 6, CH-3012, Bern, Switzerland; 3 Genetic Diversity Centre (GDC), ETH Zurich, CH-8092, Zurich, Switzerland; 4 Parallel Computing and Complex Systems Group, Department of Computer Science, University of Leipzig, Johannisgasse 26, D-04009, Leipzig, Germany; 5 Microsynth AG, Schützenstrasse 15, CH-9436, Balgach, Switzerland

## Abstract

**Background:**

Mitochondrial (mt) markers are successfully applied in evolutionary biology and systematics because mt genomes often evolve faster than the nuclear genomes. In addition, they allow robust phylogenetic analysis based on conserved proteins of the oxidative phosphorylation system. In the present study we sequenced and annotated the complete mt genome of *P. subalpina*, a member of the *Phialocephala fortinii* s.l. *– Acephala applanata* species complex (PAC). PAC belongs to the Helotiales, which is one of the most diverse groups of ascomycetes including more than 2,000 species. The gene order was compared to deduce the mt genome evolution in the Pezizomycotina. Genetic variation in coding and intergenic regions of the mtDNA was studied for PAC to assess the usefulness of mt DNA for species diagnosis.

**Results:**

The mt genome of *P. subalpina* is 43,742 bp long and codes for 14 mt genes associated with the oxidative phosphorylation. In addition, a GIY-YIG endonuclease, the ribosomal protein S3 (*Rps3*) and a putative N-acetyl-transferase were recognized. A complete set of tRNA genes as well as the large and small rRNA genes but no introns were found. All protein-coding genes were confirmed by EST sequences. The gene order in *P. subalpina* deviated from the gene order in *Sclerotinia sclerotiorum*, the only other helotialean species with a fully sequenced and annotated mt genome. Gene order analysis within Pezizomycotina suggests that the evolution of gene orders is mostly driven by transpositions. Furthermore, sequence diversity in coding and non-coding mtDNA regions in seven additional PAC species was pronounced and allowed for unequivocal species diagnosis in PAC.

**Conclusions:**

The combination of non-interrupted ORFs and EST sequences resulted in a high quality annotation of the mt genome of *P. subalpina,* which can be used as a reference for the annotation of other mt genomes in the Helotiales. In addition, our analyses show that mtDNA loci will be the marker of choice for future analysis of PAC communities.

## Background

*Phialocephala fortinii* s.l., an anamorphic ascomycete [1,2] belonging to the Helotiales [2,3], has been identified as an ubiquitous colonizer of woody plant roots colonizing up to 90% of the roots of woody plant species [4]. The geographical distribution of PAC species ranges from polar regions [5], over temperate regions [6], to subtropical regions [7]. *Phialocephala fortinii* s.l. was shown to be composed of at least 21 reproductively isolated lineages, eight of which were formally described [4,8]. In addition, a closely related but sterile species also colonizing roots of woody plant species endophytically was described as *Acephala applanata*[9]. These species are also known as *Phialocephala fortinii* s.l. – *Acephala applanata* species complex (PAC). PAC species form communities of up to 10 sympatrically occurring species and communities were shown to remain stable for several years [10-12]. No distance-decay relationship was observed among PAC communities collected across the Northern hemisphere and therefore the Baas-Becking hypothesis that “everything is everywhere” could not be rejected for this assemblage of species [4]. Although sequencing of the internal transcribed spacer (ITS) regions of the rDNA is often regarded as a ‘gold standard’ in species diagnosis in fungi [13], the resolution of ITS sequences was not sufficient to differentiate species in this complex [10]. Instead, several classes of molecular markers were developed for PAC species assignment including PCR fingerprinting, single-copy restriction fragment length polymorphisms (RFLP), multilocus sequence typing, and microsatellites. Although each of these molecular markers supported the delineation of multiple species in this complex, with concordant cryptic species defined by all markers [14,15], the application of these markers is laborious. In addition, introgression or incomplete lineage sorting further complicates species diagnosis in this species complex [14]. A single, short sequence that allows unequivocally diagnosing PAC species, and if possible other closely related taxa, is still missing.

Mitochondrial (mt) markers were successfully applied in evolutionary biology and systematics [16-18] because mt genomes often evolve faster than the nuclear genomes especially in intergenic regions [19,20]. The mt genomes of filamentous ascomycetes range between 24 to over 100 kb and have a circular topology. They usually contain 14 mt genes encoding proteins of the oxidative phosphorylation system (OXPHOS), the large (*rnl*) and small (*rns*) ribosomal subunits, and a varying set of tRNAs genes [21,22]. In addition, a varying number group I and group II introns specific to fungal mt genomes often including GIY-YIG or LAGLIDAGD endonucleases were reported [23-26] and several open reading frames with unknown functions were described [20,23,27]. In Pezizomycotina, completely sequenced and annotated mt genomes are available for members of the Eurotiomycetes and Sordariomycetes but only partial non-annotated genomes or draft annotations are available for helotialean species. The Helotiales is one of the most diverse fungal orders and is comprised of more than 350 genera including over 2,000 described species including many important plant pathogens [28].

In the course of a genome sequencing project of *P. subalpina* a draft sequence of the mt genome became available. We used this draft sequence and re-sequenced the complete mt genome of *P. subalpina.* Specifically we aimed to: (i) present a completely sequenced and annotated mt genome for the helotialean branch of the fungal tree of life, (ii) compare gene orders of *P. subalpina* with those found in other filamentous ascomycetes, (iii) compare the evolution of mt and nuclear genomes in PAC species and, (v) test mt loci as a tool for species diagnosis in PAC.

## Results

### Genome content and genome organization

**Figure 1:**
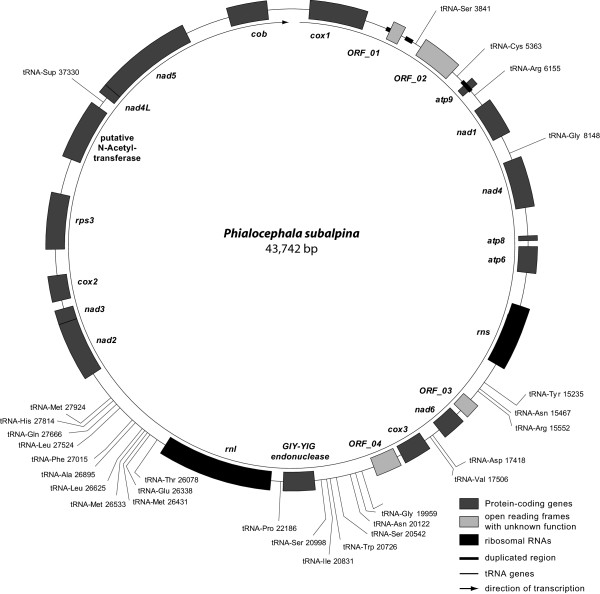
**Map of the mt genome of*Phialocephala subalpina.*** Map displaying the circular mt genome of *P. subalpina* strain UAMH 11012. All open reading frames, tRNA genes and the large ribosomal RNA are transcribed clockwise.

The circular mt genome of *P. subalpina* strain UAMH 11012 is 43,742 bp long [GenBank: JN031566] with an AT-content of 72% and contains 21 open reading frames, 14 of which code for OXPHOS proteins (*atp6*, *atp8*, *atp9*, *cox1-3*, *cob*, *nad1-6*, *nad4L*; Figure 1). All 21 protein-coding ORFs are transcribed in the same direction and start with the canonical translation initiation codon ATG except *cox1* (TTG) and *cox3* (GTG). The preferred stop codon was TAA with the exception of *nad3* and *nad5* (both TAG). Beside the 14 OXPHOS genes, the ribosomal protein S3 (*Rps3*) and a GIY-YIG endonuclease were recognized. In addition, a putative N-acetyl-transferase with an Acetyltransf_1 domain at the C-terminus was predicted (Pfam Nr: PF00583, amino acid positions 361–526, E-value: 1.8E-23). blastp searches returned several hits for Acetylase_1 containing proteins in other ascomycetes. However, all of these proteins were located in the nuclear genome. Similarly, tblastn searches of the putative mt N-acetyl-transferase against a draft genome sequence of *P. subalpina* recognized two additional N-acetyl-transferases for *P. subalpina* but both nuclear proteins were substantially smaller (198 and 202 aa) than the predicted mt protein (579 aa). The functions of the remaining four ORFs are unknown because neither significant blastp hits nor conserved domains in interproscan searches were found. In addition, the large and small ribosomal RNA subunits (*rns* and *rnl*) were present in the mt genome. A number of 5,903 Roche/454 GS FLX (454) EST sequencing reads (total number of aligned bases: 1.89 Mbp) mapped to the mt genome. Reads almost exclusively mapped to coding regions of the mt genome (proteins and rRNA genes) and all ORFs except *ORF_03* were partially or fully covered by ESTs. However, sequence coverage differed considerably among genes (see Additional file 1).

In total, 27 tRNA genes coding for all amino acids were predicted in the mt genome. Both software tools applied resulted in the same tRNA predictions except *tRNA-Cys* at position 5,363, which was only predicted by rnaweasel and *tRNA-Asn* at position 20,122 that was exclusively predicted by trnascan-se. A putative Ochre suppressor tRNA gene (antisense codon: UUA) was predicted by trnascan-se. Protein-coding genes covered 48.1% of the mt genome, while 4.7% and 11.8% correspond to tRNA genes and rRNA genes, respectively (total coding regions: 64.6%).

### Duplication of a region including the *atp9* open reading frame in PAC

**Figure 2:**
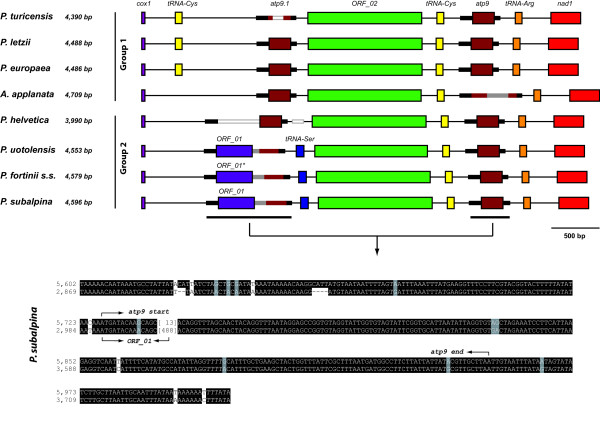
**Genome organisation of the region between*cox1*and*nad1*for eight PAC species.** Genome organisation for 8 PAC species between *cox1* and *nad1* covering the duplication, which includes the *atp9* ORF. Boxes represent ORFs and tRNA genes. Thick lines represent the position of the duplicated region and indels (black: duplicated regions upstream and downstream the *atp9* open reading frame; maroon: region of the *atp9* ORF; white: deletions; grey: inserts). The duplication including the *atp9* open reading frame was present in all species at the nucleotide level, although some species included inserts (e.g. *Acephala applanata*) or deletions (e.g. *Phialocephala turicensis*). *ORF_01* of *Phialocephala fortinii* s.s. includes a premature stop codon indicated by an asterisk. An example of an alignment for the duplicated region in *P. subalpina* is given below the overview. The intron is removed and its length was indicated in brackets. Start and stop positions of *atp9* and *ORF_01* are indicated by arrows. Numbers beside the alignment represent the mt genome coordinates for *P. subalpina.*

A duplication of an mtDNA region including the *atp9* open reading frame was observed in *P. subalpina* (Figure 1)*.* Beside the *atp9* ORF (225 bp), the duplication included 124 bp upstream of the start codon and 37 bp downstream of the stop codon of the *atp9* ORF (total length of duplication: 386 bp). The duplication was interrupted by a 488 bp insertion (Figures 1 and 2). The putative *ORF_01* (123 aa) shared the first 14 bp of the duplicated *atp9* ORF but the remaining 358 bp of *ORF_01* including the stop codon lie in the 488 bp insertion interrupting the duplication (Figure 2, bottom).

Analysis of the *cox1nad1* region for seven additional PAC strains (Table 1) showed that the synteny between *ORF_02* and *nad1* was conserved among all PAC species and sequence identity was > 90%. In contrast, it was not possible to derive significant alignments based on nucleotide data for all 8 species between *cox1* and *ORF_02*. Two groups were recognized that produced significant alignments over the entire *cox1nad1* sequences (Figure 2). Whereas the first group is comprised of *P. turicensisP. letziiP europaea* and *A. applanata*, the second group included *P. helveticaP. uotolensisP. subalpina* and *P. fortinii* s.s. The differences between *cox1* and *ORF_02* for the two groups are also reflected by the annotation of different gene features between these two groups. Species in group 1 possess an additional *tRNA-Cys* between *cox1* and *ORF_02* except for *A. applanata* and no insert was observed in the duplicated region including the *atp9* ORF. The *tRNA-Cys* was not recognized by rnaweasel in *A. applanata* due to the accumulation of mutations in this particular region compared to the other species of group 1. In contrast, members of group 2 possess a *tRNA-Ser* between *ORF_01* and *ORF_02*, which was, however, not present in *P. helvetica* due to a 118 bp deletion (Figure 2). In addition, *P. helvetica* deviated from other group 2 species by the absence of the 488 bp insertion leading to the annotation of *ORF_01*. Genome organisation between *cox1* and *nad1* reflected the known phylogenetic relatedness of PAC species with the exception of *P. uotolensis*. Strain 5_134_3 of *P. uotolensis*, which is closely related to *P. turicensis* based on sequences of several nuclear loci, single-copy RFLP analysis and microsatellite data [14,15], showed a similar genome organization as *P. fortinii s.s*. and *P. subalpina*.

**Table 1:** **PAC strains included to study the duplication of the*atp9*region and the conservation of putative ORFs**

**Taxon**	**Strain**	**Culture collection number**^**1**^	**Host**	**Geographic origin**	**GenBank accession numbers**	
					***cox1 - nad1***^**3**^	***ORF_03***^**3**^
*Phialocephala turicensis*	1_124_1	CBS 119234	*Picea abies*	Zürichberg; Switzerland	[GenBank:JN091443]	[GenBank:JN091488]
*Phialocephala letzii*	2_120_3	CBS 119266	*P. abies*	Zürichberg; Switzerland	[GenBank:JN091444]	[GenBank:JN091489]
*Phialocephala europaea*	3_117_2	CBS 119269	*P. abies*	Zürichberg; Switzerland	[GenBank:JN091445]	[GenBank:JN091490]
*Phialocephala helvetica*	4_123_4	CBS 119272	*P. abies*	Zürichberg; Switzerland	[GenBank:JN091446]	[GenBank:JN091491]
*Phialocephala uotiloensis*	5_134_3		*P. abies*	Zürichberg; Switzerland	[GenBank:JN091447]	[GenBank:JN091492]
*Phialocephala subalpina*	6_70_1	UAMH 11012	*P. abies*	Bödmeren; Switzerland	[GenBank:JN031566]	[GenBank:JN031566]
*Phialocephala fortinii* s.s.	7_6_7v		*Vaccinium myrtillus*	Bödmeren; Switzerland	[GenBank:JN091448]	[GenBank:JN091493]
*Acephala applanata*	T1_50_2		*P. abies*	Bödmeren; Switzerland	[GenBank:JN091449]	[GenBank:JN091494]

Strains of cryptic species of *Phialocephala fortinii* s.l. and *Acephala applanata* included to study the duplication of the *atp9* region and the conservation of putative ORFs

^1^CBS, Centralbureau voor Schimmelcultures, Utrecht, The Netherlands; UAMH, University of Alberta, Microfungus Collection and Herbarium, Alberta, Canada

The presence of a duplication of the region including the *atp9* ORF could be confirmed for all eight genomes at the nucleotide level (Figure 2). Three species had two non-interrupted ORFs for the *atp9* gene (*P. letzii*, *P. europaea*, *P. helvetica*) with identical protein sequences for *P. letzii* and *P. europaea*. The two *P. helvetica atp9* protein sequences deviate at two of the 74 aa positions between the two *atp9* ORFs. All other species have either an insertion leading to *ORF_01* (*P. uotolensis*, *P. fortinii* s.s., *P. subalpina*) or a deletion (*P. turicensis*) in one of the *atp9* ORFs (Figure 2). Similarly, *A. applanata* possessed an insertion of 234 bp in one of the *atp9* ORFs (Figure 2). The insert shows neither direct nor inverted repeats and did not show any similarities with known nucleotide or protein sequences using blastn and blastx searches.

### Conservation of putative ORFs in PAC species

In order to collect further evidence that putative ORFs code for proteins, the conservation of these ORFs was investigated in seven additional PAC species. Unfortunately, repeated attempts to amplify the region of *ORF_04* failed. In contrast, we could assess the presence and conservation of the other 3 putative ORFs observed in *P. subalpina* in the additional PAC species. *ORF_02* was observed in all eight PAC species. Despite SNPs and indels, none of the indels resulted in frameshift mutations and no premature stop codons were present (see Additional file 2). In contrast, *ORF_03* was either missing completely, contained stop codons, or the start codon was missing in five of the eight species. Similarly, *ORF_01* was only present in three of the eight species and *P. fortinii* s.s. had a premature stop codon (Figure 2).

### Evolution of gene orders in the Helotiales and Pezizomycotina

**Figure 3:**
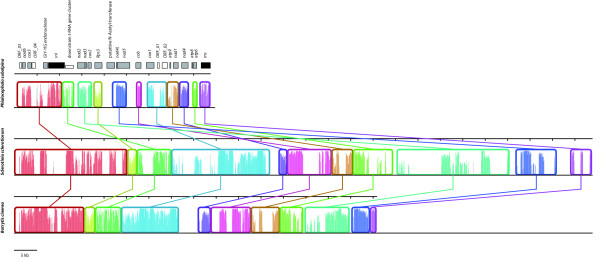
**Genome rearrangements observed among three helotialean species.** Genome rearrangements detected by Mauve genome alignments for the three helotialean species *Phialocephala subalpinaSclerotinia sclerotiorum* and *Botrytis cinerea*. Locally collinear blocks identified by mauve are given in different colours and were compared with the annotated gene features in *P. subalpina*. The *Rps3* forms a free-standing ORF in *P. subalpina* and was not placed in a group-I intron located in the U11 domain of *rnl*[26]. The *rns* gene was used as anchor to linearize the three genomes.

**Figure 4:**
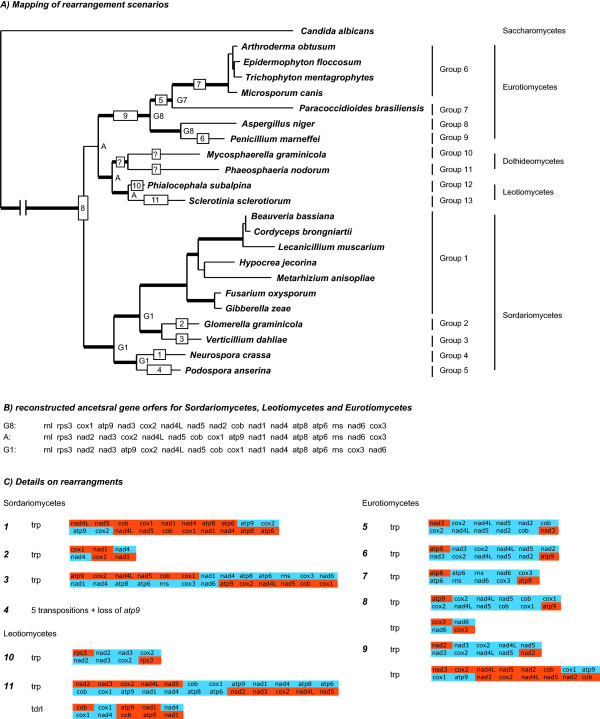
**Evolution of gene orders in Pezizomycotina.** A: Single ML tree (log likelihood: -50,671.86) constructed from 12 OXPHOS proteins for selected ascomycetes with complete mt genomes (see Table 2). The tree was used to map gene order rearrangements of 14 OXPHOS proteins, *Rps3*, and the rRNAs. Thick branches indicate bootstrap support values ≥90% in ML analysis and posterior probabilities ≥0.95 in BI. *Candida albicans* served as outgroup. Group numbering given on the right indicates groups of species with identical gene orders for the gene set analyzed. Ancestral gene orders are indicated next to the nodes as G followed by the group index if the gene order is identical to the gene order of a given group or as A for the gene order reconstructed by treerex (see B), except for the root node. Boxes on the braches indicate mapped rearrangements as analysed by crex and treerex. Numbers in boxes refer to the rearrangement given in C. B: Reconstructed ancestral gene orders for the Eurotiomycetes, Sordariomycetes and the Leotiomycetes. C: Evolutionary scenarios to deduce one gene order of the other for each change indicated in Figure 4A are given (trp, transpositions; tdrl, tandem-duplication-random-loss). Only the mt regions included in the operation are indicated in the Figure.

Anchored genome alignments for the three helotialean species *P. subalpina*, *S. sclerotiorum* and *B. cinerea* show pronounced genome rearrangements between *P. subalpina* and the closely related species *S. sclerotiorum* and *B. cinerea* (Figure 3). In contrast, complete synteny in gene order were evident between *S. sclerotiorum* and *B. cinerea*. Phylogenetic analysis based on 12 OXPHOS protein sequences (*atp6*, *cox1-3*, *cob*, *nad1-6*, *nad4L)* allowed for the identification of the four classes of filamentous ascomycetes (Figure 4, Table 2). Although the ML tree shows that the Dothideomycetes and Leotiomycetes share a most recent common ancestor with the Eurotiomycetes, the BS support value (53%) and bayesian PP value (0.55) for this placement are low. Therefore, alternative hypotheses such as Dothideomycetes and Leotiomycetes sharing a most recent common ancestor with the Sordariomycetes cannot be ruled out.

**Table 2:** Ascomycetous species included in gene order analysis

**Species**	**Class**	**Order**	**Length**	**Accession Nr**
*Aspergillus niger*	Eurotiomycetes	Eurotiales	31,103	[GenBank:NC_007445]
*Penicillium marneffei*	Eurotiomycetes	Eurotiales	35,438	[GenBank:NC_005256]
*Arthroderma obtusum*	Eurotiomycetes	Onygenales	24,105	[GenBank:NC_012830]
*Epidermophyton floccosum*	Eurotiomycetes	Onygenales	30,910	[GenBank:NC_007394]
*Microsporum canis*	Eurotiomycetes	Onygenales	23,943	[GenBank:NC_012832]
*Paracoccidioides brasiliensis*	Eurotiomycetes	Onygenales	71,335	[GenBank:NC_007935]
*Trichophyton mentagrophytes*	Eurotiomycetes	Onygenales	24,297	[GenBank:NC_012826]
*Mycosphaerella graminicola*	Dothideomycetes	Capnodiales	43,964	[GenBank:NC_010222]
*Phaeosphaeria nodorum*	Dothideomycetes	Pleosporales	49,761	[GenBank:EU053989]
*Botrytis cinerea*	Leotiomycetes	Helotiales	80,799	[GenBank:NW_001814287]^1^
*Phialocephala subalpina*	Leotiomycetes	Helotiales	43,742	[GenBank:JN031566]
*Sclerotinia sclerotiorum*	Leotiomycetes	Helotiales	128,852	draft^2^
*Beauveria bassiana*	Sordariomycetes	Hypocreales	29,961	[GenBank:NC_010652]
*Cordyceps brongniartii*	Sordariomycetes	Hypocreales	33,926	[GenBank:NC_011194]
*Fusarium oxysporum*	Sordariomycetes	Hypocreales	34,477	[GenBank:AY945289]
*Gibberella zeae*	Sordariomycetes	Hypocreales	95,676	[GenBank:NC_009493]
*Hypocrea jecorina*	Sordariomycetes	Hypocreales	42,130	[GenBank:NC_003388]
*Lecanicillium muscarium*	Sordariomycetes	Hypocreales	24,499	[GenBank:NC_004514]
*Metarhizium anisopliae*	Sordariomycetes	Hypocreales	24,673	[GenBank:NC_008068]
*Glomerella graminicola*	Sordariomycetes	n.a.	39,649	[GenBank:CM001021]
*Verticillium dahliae*	Sordariomycetes	Phyllachorales	27,184	[GenBank:NC_008248]
*Neurospora crassa*	Sordariomycetes	Sordariales	64,840	draft^2^
*Podospora anserina*	Sordariomycetes	Sordariales	94,192	[GenBank:NC_001329]
*Candida albicans*	Saccharomycetes		40,420	[GenBank:NC_002653]

^1^partial sequence (without annotation)

^2^sequence and annotation derived from http://www.broadinstitute.org

Based on this phylogeny, we have reconstructed putative ancestral gene orders and rearrangements leading to the contemporary gene orders of 14 OXPHOS proteins (*atp6, atp8, atp9*, *cox1-3*, *cob*, *nad1-6*, *nad4L)*, *Rps3,* and rRNAs by using crex and treerex analysis (Figures 4A, B; see Additional file 3 for full input gene orders). Identical ancestral gene arrangements were observed in treerex analysis irrespective whether the Leotiomycetes and Dothideomycetes share the most recent common ancestor with the Sordariomycetes or the Eurotiomycetes (data not shown). The evolution of gene orders among species belonging to the Pezizomycotina was mainly characterized by transpositions (Figures 4A, C). In contrast, inversions and inverse-transpositions have not been found in the analysed gene orders, which excluded the two Dothideomycete species. However, the Dothideomycetes have genes that are in opposite orientation with respect to the remaining Pezizomycotina species. Moreover, both species were separated from all other Pezizomycotina species and by themselves by very long evolutionary scenarios rendering the reconstruction unreliable (Figure 4A). In our analysis, only one tandem-duplication-random-loss (trdl) event was suggested by crex analysis involving *cob* and *cox1* to derive the gene order of *S. sclerotiorum* (Rearrangement 11 in Figure 4). blastx searches of *cob* and *cox1* protein sequences against the mtDNA of *S. sclerotiorum* confirmed that a duplication-based rearrangement mechanism was involved because a partial *cob* fragment in front of *cox1* was found as expected for the predicted operation (30 aa, E-value: 3.00E-09). Together with the duplication of *atp9* for PAC species (see above) this indicates that duplication-based rearrangement are involved in mitogenome evolution of Pezizomycotina. The number of necessary events required to convert one gene order to the other was not always related to the phylogenetic distance between species. For example, gene orders of *Podospora anserina* and *S. sclerotiorum* could only be derived from gene orders of their closest relatives by evolutionary scenarios that include multiple transpositions and in the case of *S. sclerotiorum* a tdrl event.

Based on the used species and gene sampling our analysis indicates that i) the group of *Beauveria bassiana* (Group 1 in Figure 4) represent the ancestral gene order for the Sordariomycetes, i.e., no gene rearrangements are necessary to reach the node in the phylogenetic tree representing the Sordariomycetes. Similarly, *Aspergillus niger* (Group 8 in Figure 4) represents the ancestral gene order for the Eurotiomycetes. Furthermore, comparisons among the possible intermediate gene orders of the Sordariomycetes, Eurotiomycetes, and the reconstructed ancestral gene order of the Leotiomycetes indicates the intermediate position of the later (Additional file 4).

### Evolution of mtDNA in PAC and searching regions for species diagnosis

**Figure 5:**
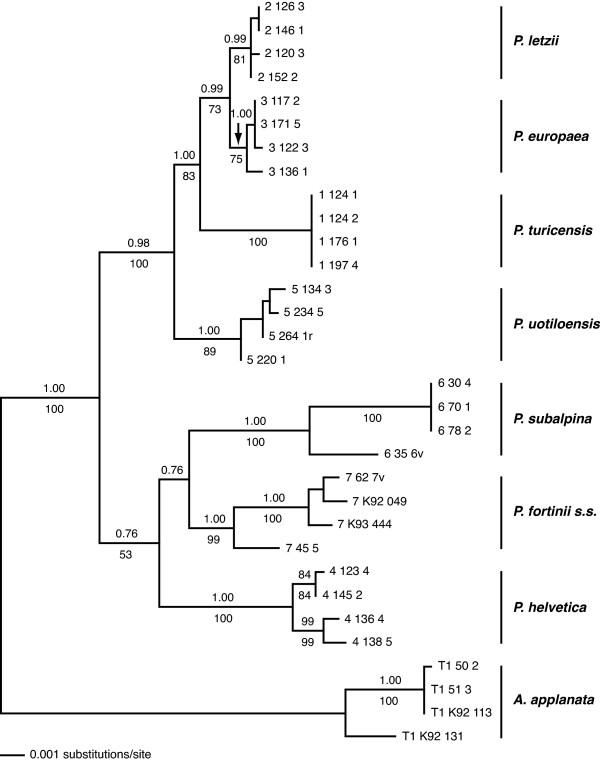
**Phylogenetic analysis for PAC species based on three mt loci.** Single ML tree for eight PAC species based on DNA sequence data of three concatenated mt loci. Posterior probabilities of BI (above branches) and bootstrap values of ML analysis (below branches) are indicated. *Acephala applanata* was chosen as outgroup.

A total of 7,350 bp (16.8% of the mt genome) from four regions of the mt genome were amplified in four additional PAC species to screen for polymorphisms (Table 3). Fragments included coding (*cox1rnl*) and intergenic regions (*Rps3-*putative N-acetyl-transferase, *atp9-nad1-nad4*). High amounts of DNA polymorphisms were observed within coding and intergenic regions among PAC species (Additional file 5). However, DNA polymorphisms were not always evenly distributed among species within the sequenced DNA fragments. Whereas in three cases all five species contributed to a similar extent to the observed polymorphic regions (e.g. *rnl*), *P. europaea* was almost exclusively responsible for a peak in diversity observed in *cox1* (data not shown). In addition, a 520 bp indel in the non-coding region spanning *Rps3* and the putative *N-acetyl-transferase* showed a very high nucleotide diversity among the three species with this insert (*P. europaeaP. fortinii* s.s., *A. applanata*) (Additional File 5). Based on these results three regions were selected and tested on a broader selection of strains. Parts of *rnl* (~730 bp), a fragment located between *Rps3* and the putative N-acetyl-transferase (~850 bp), and a fragment including parts of *nad1* and *nad4* (~1,650 bp) were sequenced from 32 strains belonging to eight PAC species (Table 3). Best-fitting mutation models selected from jModeltest were F81 + I, TVM + G, TrN + G, and TPM3uf + I + G for the three individual loci and the concatenated dataset, respectively. Variation in the three fragments was considerable among and within species. Often single strains of a species were responsible for this high variation found within species (Figure 5). For example, strains 7_45_5, or T1_K92_131 were separated from the other strains of the respective species for all three loci resulting in 19 and 21 substitutions respectively, not considering indels. *P. turicensis* was the only species that was monomorphic for all three loci. ML and BI analysis using the concatenated dataset placed strains of each species in well-supported clades (Figure 7). In addition, the tree topology of each individual mtDNA locus and the concatenated mtDNA dataset did not deviate significantly from the topology of the concatenated sequences of four genomic regions sequenced previously [14] (Table 4). No evidence for recombination blocks among the three sequenced loci were found, although incompatible sites were regularly observed (data not shown). Incompatible sites were located within as well as between the three sequenced loci.

**Table 3:** PAC strains included to study the suitability of mtDNA markers for species diagnosis

**Taxon**	**Strain**^1^	**Culture collection number**^2^	**Host**	**Geographic origin**	**GenBank accession numbers**			
					***cox1***^3^	***rnl***	***Rps3 - N-acetyl- transferase***	***atp9 - nad4***
*Phialocephala turicensis*	1_124_1	CBS 119234	*Picea abies*	Zürichberg; Switzerland	n.a.	[GenBank:JN091454]	[GenBank:JN091495]	[GenBank:JN091530]
	1_124_2		*P. abies*	Zürichberg; Switzerland	n.a.	[GenBank:JN091455]	[GenBank:JN091496]	[GenBank:JN091531]
	1_176_1*	CBS 119264	*P. abies*	Zürichberg; Switzerland	n.a.	[GenBank:JN091456]	[GenBank:JN091497]	[GenBank:JN091532]
	1_197_4	CBS 119265	*P. abies*	Zürichberg; Switzerland	n.a.	[GenBank:JN091457]	[GenBank:JN091498]	[GenBank:JN091533]
*Phialocephala letzii*	2_120_3	CBS 119266	*P. abies*	Zürichberg; Switzerland	n.a.	[GenBank:JN091458]	[GenBank:JN091499]	[GenBank:JN091534]
	2_126_3		*P. abies*	Zürichberg; Switzerland	n.a.	[GenBank:JN091459]	[GenBank:JN091500]	[GenBank:JN091535]
	2_146_1*	CBS 119268	*P. abies*	Zürichberg; Switzerland	n.a.	[GenBank:JN091460]	[GenBank:JN091501]	[GenBank:JN091536]
	2_152_2	CBS 119267	*P. abies*	Zürichberg; Switzerland	n.a.	[GenBank:JN091461]	[GenBank:JN091502]	[GenBank:JN091537]
*Phialocephala europaea*	3_117_2	CBS 119269	*P. abies*	Zürichberg; Switzerland	n.a.	[GenBank:JN091462]	[GenBank:JN091503]	[GenBank:JN091538]
	3_122_3	CBS 119270	*P. abies*	Zürichberg; Switzerland	[GenBank:JN091450]	[GenBank:JN091463]	[GenBank:JN091504]	[GenBank:JN091529]
	3_136_1		*P. abies*	Zürichberg; Switzerland	n.a.	[GenBank:JN091464]	[GenBank:JN091505]	[GenBank:JN091539]
	3_171_5*	CBS 119271	*P. abies*	Zürichberg; Switzerland	n.a.	[GenBank:JN091465]	[GenBank:JN091506]	[GenBank:JN091540]
*Phialocephala helvetica*	4_123_4	CBS 119272	*P. abies*	Zürichberg; Switzerland	n.a.	[GenBank:JN091466]	[GenBank:JN091507]	[GenBank:JN091541]
	4_136_4		*P. abies*	Zürichberg; Switzerland	n.a.	[GenBank:JN091467]	[GenBank:JN091508]	[GenBank:JN091542]
	4_138_5*	CBS 119273	*P. abies*	Zürichberg; Switzerland	n.a.	[GenBank:JN091468]	[GenBank:JN091509]	[GenBank:JN091543]
	4_145_2	CBS 119274	*P. abies*	Zürichberg; Switzerland	n.a.	[GenBank:JN091469]	[GenBank:JN091510]	[GenBank:JN091544]
	4_153_2		*P. abies*	Zürichberg; Switzerland	[GenBank:JN091451]	[GenBank:JN091470]	[GenBank:JN091511]	[GenBank:JN091545]
*Phialocephala uotiloensis*	5_134_3		*P. abies*	Zürichberg; Switzerland	n.a.	[GenBank:JN091471]	[GenBank:JN091512]	[GenBank:JN091546]
	5_220_1	CBS 119276	*P. abies*	Uetliberg; Switzerland	n.a.	[GenBank:JN091472]	[GenBank:JN091513]	[GenBank:JN091547]
	5_234_5	CBS 119277	*P. abies*	Uetliberg; Switzerland	n.a.	[GenBank:JN091473]	[GenBank:JN091514]	[GenBank:JN091548]
	5_264_1r*	CBS 119275	*P. abies*	Uetliberg; Switzerland	n.a.	[GenBank:JN091474]	[GenBank:JN091515]	[GenBank:JN091549]
*Phialocephala subalpina*	6_30_4	CBS 119279	*P. abies*	Bödmeren; Switzerland	n.a.	[GenBank:JN091475]	[GenBank:JN091516]	[GenBank:JN091550]
	6_35_6v	CBS 119278	*Vaccinium myrtillus*	Bödmeren; Switzerland	n.a.	[GenBank:JN091476]	[GenBank:JN091517]	[GenBank:JN091551]
	6_70_1	UAMH 11012	*P. abies*	Bödmeren; Switzerland	[GenBank:JN031566]	[GenBank:JN031566]	[GenBank:JN031566]	[GenBank:JN031566]
	6_78_2*	CBS 119280	*P. abies*	Bödmeren; Switzerland	n.a.	[GenBank:JN091477]	[GenBank:JN091518]	[GenBank:JN091553]
*Phialocephala fortinii* s.s.	7_6_7v		*V. myrtillus*	Bödmeren; Switzerland	[GenBank:JN091452]	[GenBank:JN091480]	[GenBank:JN091519]	[GenBank:JN091554]
	7_45_5	CBS 119281	*P. abies*	Bödmeren; Switzerland	n.a.	[GenBank:JN091478]	[GenBank:JN091520]	[GenBank:JN091555]
	7_62_7v	CBS 119282	*V.myrtillus*	Bödmeren; Switzerland	n.a.	[GenBank:JN091479]	[GenBank:JN091521]	[GenBank:JN091556]
	7_K92_049	CBS 114608	*P. abies*	Odenwald; Germany	n.a.	[GenBank:JN091481]	[GenBank:JN091522]	[GenBank:JN091557]
	7_K93_444*	CBS 443.86	*P. sylvestris*	Suonenjoki; Finland	n.a.	[GenBank:JN091482]	[GenBank:JN091523]	[GenBank:JN091558]
*Acephala applanata*	T1_50_2		*P. abies*	Bödmeren; Switzerland	n.a.	[GenBank:JN091483]	[GenBank:JN091524]	[GenBank:JN091559]
	T1_51_3		*P. abies*	Bödmeren; Switzerland	n.a.	[GenBank:JN091484]	[GenBank:JN091525]	[GenBank:JN091560]
	T1_K92_113*	CBS 109321	*P. abies*	Bödmeren; Switzerland	n.a.	[GenBank:JN091485]	[GenBank:JN091526]	[GenBank:JN091561]
	T1_K92_131	CBS 109322	*P. abies*	Bödmeren; Switzerland	[GenBank:JN091453]	[GenBank:JN091486]	[GenBank:JN091527]	[GenBank:JN091562]

Strains of cryptic species of *Phialocephala fortinii* s.l. and *Acephala applanata* included to study the suitability of mtDNA markers for species diagnosis

^1^type strains are marked by an asterisk

^2^CBS, Centralbureau voor Schimmelcultures, Utrecht, The Netherlands; UAMH, University of Alberta, Microfungus Collection and Herbarium, Alberta, Canada

^3^sequences are only available for strains included in the screening to detect mtDNA polymorphisms

**Table 4:** Concordance in evolution among mtDNA and nucDNA for PAC

		**Probability p**			
Test^1^	Tree	*rnl*	*Rps3-* putative N- acetyl-transferase	*nad1*-*nad4*	concatenated
WSH	4 nucDNA loci^1^	0.652	0.346	0.561	0.750
AU	4 nucDNA loci	0.125	0.095	0.370	0.499

Comparison of tree topologies for three mtDNA loci and four concatenated nuclear loci for eight species belonging to the *Phialocephala fortinii* s.l.-*Acephala applanata* species complex. Both, the approximately unbiased (AU) and the weighted Shimodaira and Hasegawa (WSH) tests were calculated

^1^tree topologies were compared using the approximately unbiased (AU) and the weighted Shimodaira and Hasegawa (WSH) tests [29]

^2^The 4 nuclear loci included two non-coding loci (pPF-018, pPF-076) and partial sequences of two coding loci (β-tubulin, translation elongation factor 1-α) as described in Grünig et al. [14]

## Discussion

In the present study we sequenced and annotated the mt genome of a widely distributed group of root-inhabiting fungi belonging to the Helotiales (Leotiomycetes). Gene order analysis showed that the evolution of mt genomes in the Pezizomycotina is mainly driven by transpositions. Moreover, we show the usefulness of mtDNA loci for species diagnosis in the *Phialocephala fortinii* s.l. – *Acephala applanata* species complex (PAC).

### Gene content and mt organisation in *P. subalpina*

The mt genome of filamentous ascomycetes normally code for 14 proteins of the oxidative phosphorylation system [22], including the sequences for the large (*rnl*) and small (*rns*) ribosomal subunits. In addition, a varying number of additional proteins with homologies to known proteins, i.e., the ribosomal protein S3 (*Rps3*) [23,24], and ORFs with unknown functions may be found [20,23,27]. The mt genome of *P. subalpina* follows this rule. All 14 OXPHOS proteins, the two rRNAs and a complete set of tRNA genes were present. Nevertheless, some unique features were observed. (i) A putative N-acetyl-transferase was predicted in the mt genome. To the best of our knowledge, this is the first report of a putative N-acetyl-transferase in the mt genome of ascomycetes. Acetyl-transferases modify proteins in eukaryotes, both co- and post-translationally by transferring acetyl groups from acetyl-coenzyme A to either the a-amino group of amino-terminal residues or to the e-amino group of lysine residues at various positions [30]. In addition, N-acetyl-transferases were shown to modify several ribosomal proteins in *Escherichia coli*[31,32]. Although it seems likely that the putative mt N-acetyl-transferase is involved in protein modifications, the exact function is unknown because N-acetyl-transferases can act on different groups of substrates [30]. (ii) The ribosomal protein S3 (*Rps3*) formed a free-standing ORF in *P. subalpina* (Figure 1). To the best of our knowledge this is the second report of a free-standing *Rps3* gene in the Pezizomycotina. In all completely sequenced mt genomes of filamentous ascomycetes possessing *Rps3*, this gene is placed in a group-I intron located in the U11 domain of the *rnl* except for *Phaeospaeria nodorum*[23,24,26,33]. Whereas the *Rps3* of *P. nodorum* is large (771 aa) and includes parts of *cox1*[34], *Rps3* found in *P. subalpina* is similar in length as *Rps3* found in *S. sclerotiorum* and other ascomycetes. In addition, blastp searches revealed no segments of other mt proteins. *Rps3* followed *cox2* in both species and sequencing of additional mt genomes in the Dothideomycetes and Leotiomycetes will show whether *cox2Rps3* synteny may be another common position of *Rps3* in mt genomes. (iii) An Ochre suppressor tRNA gene was predicted. Suppressor tRNAs were found in the genomes of many species and allow the read-through of stop codons [35-37]. They provide a regulatory mechanism of gene expression allowing the production of several proteins from a single gene and were shown to be especially important for RNA viruses [38]. Moreover, suppressor tRNAs are hypothesized to play a role for the transcription of cryptic mitochondrial gene on the antisense strand [39,40]. (iv) A duplication of a genomic region including the *atp9* open reading frame interrupted by a 488 bp insert was observed in *P. subalpina*, leading to the annotation of *ORF_0*1. Re-sequencing of the *cox1-ORF_01-ORF_02-atp9-nad1* region for additional seven PAC species confirmed that three species have two intact ORFs coding for *atp9*. In all other species one of the two *atp9* ORFs was interrupted by indels indicating that the duplication of the *atp9* region followed by successive modifications predated the separation of PAC species. Interestingly, two types of sequences with no obvious similarity were found between *cox1* and the duplicated *atp9* regions for PAC species, which was reflected in the annotated gene features and was in accordance with the relatedness of the species. An exception was *P. uotolensis* strain 5_134_3, which showed a similar sequence and organisation as *P. subalpina* and *P. fortinii* s.s. In previous studies we showed for this strain that it might be the result of a hybridization event with *P. subalpina*. For example the same strain clustered with a *P. subalpina* strain for the sequence of the translation elongation factor 1-α [14] but three other nuclear loci, microsatellite analysis and single-copy RFLP analysis placed this strain with other strains of *P. uotolensis*[8,14,15].

### Transcription of ORFs in the mt genome

Identification of mtDNA genes is no guarantee that they are also active, as was e.g. shown for a silent copy of the *cox2* gene in the soybean mitochondrion [19,41]. Mapping of 454 EST sequence reads showed that they were mostly restricted to ORFs and rDNA genes. All ORFs except *ORF_03* were confirmed by ESTs. However, pronounced differences in the sequence coverage were observed. Several factors could explain the differences observed in the transcription profile of coding regions. Either the 454 sequencing protocol introduced non-uniform coverage in the normalized cDNA library [42] or the differences in the coverage reflect some differences in the expression of mt genes. Indeed, transcription of mt genes in the ascomycete *Saccharomyces cerevisiae* was shown to be far from uniform [43]. Future RT-qPCR assays for *P. subalpina* will show whether the observed differences in cDNA coverage reflect differences in expression profiles for mt proteins.

### Do putative ORFs code for proteins?

Putative ORFs are regularly annotated in fungal genomes [20,23,27,44]. However, whether they really code for proteins is rarely assessed. In the present study we used an indirect approach to gain additional evidence whether putative ORFs code for proteins. We assume that putative ORFs that code for a protein should (i) be transcribed, i.e., ESTs should match the respective regions and (ii) nucleotide sequences of the ORFs should be conserved among closely related species, in particular premature stop codons and frameshifts should not be detected. *ORF_02* is an example of a transcribed and conserved ORF that most likely codes for a protein although blastp searches recognized no similar proteins in the NCBI database. In contrast, *ORF_03* was either completely deleted or included premature stop codons in four species and the insert responsible for *ORF_01* was only present in three species and included a frame-shift mutation in one of these species. Therefore, we hypothesize that *ORF_01* and *ORF_03* do not code for proteins because they are not universally conserved despite their (partial) transcription (see Additional file 1). However, definitive evidence that conserved and transcribed ORFs code for proteins is only given when the corresponding proteins are isolated [19].

### Evolution of gene orders in filamentous ascomycetes

Phylogenetic analysis based on protein data of 12 OXPHOS genes resulted in a robust phylogeny with the exception of the Dothideomycetes and Leotiomycetes which clustered together, but their position was not resolved and it remained unclear whether they share the most common ancestor with the Sordariomycetes or the Eurotiomycetes. Indeed, several deep phylogenetic analysis based on nuclear genes and/or rDNA loci placed the Leotiomycetes in a cluster with the Sordariomycetes [43,44]. To attribute for the uncertainty of the placement of Leotiomycetes, we analyzed the evolution of gene orders using both alternative hypotheses.

Gene orders were compared previously for some fungal orders [16,45,46] but no automated and formally well-defined approach was used to reconstruct the rearrangement history. crex is based on the notion of common intervals, which reflect genes that appear consecutively in the input gene orders. In addition, we used treerex resulting in a detailed reconstruction of the rearrangement history and our analysis revealed that the evolution of gene orders in filamentous ascomycetes is mainly driven by transposition events and that gene orders are (mostly) conserved for close relatives. The conservation of gene orders in close relatives was also shown in previous studies. For example, the transposition leading to the gene order of *Verticillium dahliae* (Rearrangement 3 in Figure 4) was confirmed in seven additional *Verticillium* species [16]. Similarly, 3 species closely related to *A. niger* (*Aspergillus tubingensisPenicillium chrysogenum* and *Penicillium digitatum*) were recently shown to have the same gene order as *A. niger* for the set of genes used in our analysis [47].

Gene order analyses were shown to be useful to confirm phylogenetic analysis in several organismal groups [45-49] and in the present study we show, that the extent of gene rearrangements reflects the phylogenetic position of a species in most cases. For example, the ancestral gene order of the Leotiomycetes was most closely related to the two ancestral gene orders of the Sordariomycetes and Eurotiomycetes based on the parsimonious rearrangement scenarios reconstructed by crex reflecting an intermediate position of the Leotiomycetes (see Additional File 4). This fits well with the results of the phylogenetic analysis showing that the Leotiomycetes could share the most recent common ancestor either with the Sordariomycetes or the Eurotiomycetes. However, two exceptions were observed. First, *P. anserina* deviated from *N. crassa* by several transpositions and the loss of the *atp9* gene. Similarly, the two Dothideomycete species deviated from themselves and all other Pezizomycotina by complex rearrangement scenarios. It was possible to reconstruct the possible ancestral gene order for the Sordariomycetes and the Eurotiomycetes although the presented results must be regarded as preliminary because of the limited species sampling in particular in the Leotiomycetes and the exclusion of the tRNAs due to restrictions of the software tools used. Since tRNAs are important for understanding the evolution of gene orders [49,50] the presented results need to be verified with a data set including the tRNAs.

### Evolution of mtDNA in PAC species and suitability of mt markers for species diagnosis

The amount of variation found within PAC species was high, despite the fact that strains from most of the species were derived from a single study site (Table 3). Intra-species variability in the mt genome among species belonging to ascomycetes can vary greatly. Whereas mtDNA was highly diverse in many collections of filamentous ascomycetes [20,51,52], low levels of diversity were observed in *M. graminicola* for a world-wide collection of strains [27]. The high amount of variation in PAC species could be the result of genetic exchange among mt genomes. Although mt genomes were characterized by the absence of any genetic recombination in some studies [27] an increasing number of reports show recombination in mtDNA of plants and fungi [52-54]. Therefore, we searched for inconsistencies in our dataset to find evidence for recombination events. Although inconsistencies were observed among parsimony informative sites for PAC species, the inconsistencies occurred within loci as well as between loci and no recombination blocks were evident, indicating that rather parallel mutations than recombination characterize the evolution of the mt genome in PAC species.

Mt loci were often used as diagnostic markers in systematics. They received particular attention during barcoding campaigns [55] and were also successfully applied for fungal species diagnosis [18,20,56]. In the present study it was possible to analyze the resolution of mtDNA markers at a fine taxonomic level using a well-defined species complex. Several marker types were used to characterize cryptic species in PAC such as single-copy RFLPs, microsatellite markers and sequence markers [14,15]. In the present study we show that two of the three examined mtDNA regions allow to distinguish all eight PAC species. Only one strain of *P. uotolensis* was misplaced using the sequence information of the partial *rnl* gene. The amount of variation found in three mt loci was high compared to nuclear loci sequenced previously [14]. For example, *P. letzii* and *P. europaea* have identical sequences for several nuclear coding and non-coding loci and were difficult to diagnose based on single-copy RFLP markers [14] but formed well supported monophyletic groups for all mt loci. So far, mating type idiomorphs were the only nuclear regions with a similar ability to distinguish PAC species [57]. However, a disadvantage of the mating type for species diagnosis is their heterothallic organisation in PAC species, which does not allow amplifying a single fragment for all strains. In this respect, mt markers developed in the present study offer advantages and mt loci can be regarded as useful diagnostic markers for PAC.

## Conclusions

The availability of the complete annotated mt genome and the knowledge about the intra- and inter-species diversity in PAC provides the basis for the development of new markers to study the community ecology, population biology and evolution in this species complex. In addition, it provides a reference for the annotation of other mt genomes in the Helotiales.

## Methods

### Sequencing the complete mt genome of *Phialocephala subalpina*

In the course of a genome sequencing project of *P. subalpina* strain UAMH 11012, an initial Roche/454 GS FLX (454) shotgun run was performed at the Functional Genomics Centre Zurich (FGCZ, Uni/ETH Zurich) and from that a draft of the circular mt genome of *P. subalpina* strain UAMH 11012 became available. The draft sequence was subdivided into 12 fragments (see Additional file 6) and amplified from strain UAMH 11012 using long-range PCR in 20 μl volumes (Expand Long Range dNTPack kit, Roche, Rotkreuz, Switzerland). PCR conditions were optimized for 4 kb fragments with high AT contents by lowering the temperature during the elongation step from 68°C to 62°C and an initial elongation time of 4 min. PCR fragments were purified using the Wizard Plus SV kit (Promega, Wallisellen, Switzerland) and sequenced at Microsynth (Balgach, Switzerland).

### Annotation of the mt genome

Open reading frames (ORFs) in the mtDNA sequence of *P. subalpina* were searched with NCBI orf finder[58]. blastp[59] and interproscan[60] were used for homology-based function prediction of proteins. In addition, homologous regions of putative ORFs were sequenced for seven PAC species and sequences were analyzed for indels and SNPs resulting in frameshift mutations and/or pre-mature stop codons. The tRNA genes were predicted by trnascan-se v1.21[61] and rnaweasel[62] searches using default settings for mt genomes. The ribosomal RNAs were determined by comparison with sequences from other fungi using blastn. Group I and group II introns were predicted using rnaweasel[62]. Duplications in the mt genome were searched using the software dotter[63].

### EST evidence for ORFs

Expression of ORFs was tested by searching for ESTs derived from a normalized EST library of strain UAMH 11012 sequenced on the 454 platform at FGCZ (Rémy Bruggmann, unpublished). Single 454 cDNA reads belonging to the mt genome were filtered using the software genomethreader[64] and the sequence coverage for each base in the mt genome was calculated (= number of reads covering a specific base in the genome).

### Analyzing the duplication of the *atp9* region in PAC species

A region between *cox1* and *nad1* included a duplication of the *atp9* region in *P. subalpina* (see results). In order to test the presence of the duplication in other PAC species, the region between *cox1* and *nad1* was sequenced from additional 7 PAC species (Table 1). The entire locus was amplified using primers mtPF_F07_F27774 (TAGAGGTAATCAAACCAATG) and PF_cox1_F (AGCCCACCAAAACCTCATGC) using long-range PCR and sequenced as described above.

### Evolution of gene orders in Pezizomycotina

In a first step, mt genome alignments for the helotialean species *B. cinereaS. sclerotiorum* and *P. subalpina* based on the publically available sequences were performed using the mauve 2.3.1 software [65] and locally collinear blocks identified by mauve were compared with the annotated gene features in *P. subalpina*. In a second step, the phylogenetic relationship among species belonging to the Pezizomycotina was analyzed using a concatenated protein dataset of 12 OXPHOS proteins (*atp6cox1-3cobnad1-6nad4L)*. Proteins coding for *atp8* (48 aa) and *atp9* (74 aa) are missing in one or more of the species included in the analysis (*atp8*: *P. nodorum; atp9*: *P. anserina* and *P. nodorum*) and we omitted these two genes in the phylogenetic analysis. The protein data set was aligned using mafft[66] and the mafft-homologues option was applied. The resulting alignment was trimmed in gblocks 0.91b[67] using default settings. *Candida albicans* served as outgroup in the phylogenetic analysis (see below).

In a third step, gene order of all 14 OXPHOS proteins, *Rps3,* and rRNAs (*rns* and *rnl*) was studied for 22 species in Pezizomycotina (Table 2) using crex[68] and treerex[69] analysis. A limitation that applies to most gene order analysis approaches, including crex and treerex is that they can only be applied to gene orders with an equal gene content with each gene appearing exactly once. Therefore, we had to exclude tRNA genes because the identification of homologous tRNA genes was hampered in our dataset due to gene losses and/or multiple occurrences of the same tRNA genes (see Additional file 7). Moreover, tRNAs are difficult to predict by the available tools as we have shown for *P. subalpina* (see Results), rendering them uncertain candidates for gene order analysis. An informal introduction to crex and treerex analysis as well as a detailed description of the performed gene order analysis is given in the Additional file 4. The Dothideomycetes were excluded from analysis because both species were separated from all other Pezizomycotina and by themselves by very long evolutionary scenarios including several inversion and tdrl events which makes the reconstruction unreliable. Similarly, the gene order of *P. anserina* was excluded since TreeREx cannot handle gene orders with unequal gene content (loss of *atp9* in *P. anserina*). The scenario shown in Figure 4 is derived from the separate analysis of *N. crassa* and *P. anserina* considering the common genes only.

### Searching regions for species diagnosis in PAC

The following strategy was applied to identify regions suitable for species diagnosis in PAC. In a first step, four fragments each between 1,200-2,800 bp long spanning coding and intergenic regions in the mtDNA were chosen (*cox1rnlnad1-nad4Rps3-* putative N-acetyl-transferase) and amplified in four additional PAC species (*A. applanataP. fortinii* s.s., *P. helvetica*, and *P. europaea*) including the phylogenetically most distantly related PAC species known to date (*P. europaea* and *A. applanata*) [14]. Coding regions were chosen because they were either shown to be suitable for species diagnosis previously [17] or have the potential to be used to diagnose also closely related species of PAC. In contrast, intergenic regions were chosen because they were shown to be highly polymorphic [20]. Nucleotide diversity for the five species was analyzed in dnasp v5.0[70] and sites[71]. Based on these results, one coding region (*rnl*) and two intergenic regions (*nad1-nad4Rps3-* putative N-acetyl-transferase) were selected. These three loci were then tested on a broader collection of strains to study the evolution of mtDNA within PAC species and their suitability for species diagnosis. The dataset includes 32 strains belonging to 8 PAC species (Table 3). For primers used to amplify and sequence fragments see Additional file 8. Fragments were amplified in a 15 μl reaction volume using approximately 5 ng of template DNA. After an initial denaturation step for 2 min at 94°C, 31 cycles were performed each consisting of a denaturation step at 94°C for 30 s, an annealing step at 50°C for 30 s and an extension step at 60°C for 90 s [72] followed by a final extension step for 6 min at 72°C. Fragments were directly purified using an ExoSap protocol [15] and sequencing was conducted at the Genetic Diversity Centre (GDC, ETH Zurich). The nucleotide sequences of each locus were aligned with ClustalW separately and the resulting data matrices were then concatenated into one combined data matrix and subjected to phylogenetic analysis.

### Phylogentic analysis

Phylogenetic trees were inferred using Bayesian inference (BI) and maximum likelihood (ML) methods for the protein and nucleotide dataset. ML analysis for the protein dataset was performed in treefinder[73] using the substitution model selected by protest[74]. ML analysis for nucleotide datasets were performed in paup[75] using the substitution model selected by jmodeltest v1.0[76]. Branch supports were provided by 1,000 bootstrap replicates for both datasets. BI trees were calculated with mrbayes 3.1[77] for protein and nucleotide datasets by running two analyses each consisting of two simultaneous runs with four heated chains per run. Each analysis was run for 5 Mio generations and trees were sampled every 100 generations. Post-burn-in was assumed when the average standard deviation of split frequencies was consistently ≤ 0.01. Post-burn-in trees were collected and the parameter and topology summarizations calculated. To ensure that the analyses reached stationarity and converged on the same topology and likelihood scores, the resulting likelihoods, tree topologies and model estimates were compared by eye.

The tree topologies derived from the nucleotide data sets (each single locus and the concatenated alignment) were compared with tree topologies from the concatenated sequences of four genomic regions (*b-tubulinelongation factor 1-αpPF_018* and *pPF_076*) sequenced previously [14] using the approximately unbiased (AU) and the weighted Shimodaira and Hasegawa (WSH) tests [29]. Only one strain (type strain) per species was included to compare tree topologies. Both tests were calculated in treefinder using 10,000 replicates. In addition, a compatibility matrix of nucleotide substitutions was constructed using the software Sites[71] to search for possible recombination blocks in the datasets.

## Abbreviations

PAC: Phialocephala fortinii s.l. – Acephala applanata species complex; OXPHOS: Proteins of the oxidative phosphorylation system; ORF: Open reading frame.

## Competing interests

The authors declare that they have no competing interests.

## Authors’ contributions

AD and CRG designed the research project. AD and CRG performed all experiments and collected sequence data. RB, SZ, MB and CRG implemented analytical tools and performed analysis. CRG, RB, MB and SZ wrote the manuscript. All authors have read and approved the final manuscript.

## Supplementary Material

Additional file 1 Titel: Transcriptomics landscape in the mt genome of *Phialocephala subalpina*. Description: Sequence coverage for 5,903 454 cDNA reads (total number of aligned bases: 1.89 Mbp) that mapped to the mt genome of *Phialocephala subalpina*. 454 reads almost exclusively mapped to coding regions of the mt genome (proteins and rDNAs) but depths of coverage differed considerably among genes. Click here for file

Additional file 2 Titel: Protein aligenemts for putative ORFs. Description: Protein alignments of two putative ORFs (*ORF_02, ORF_03)* for eight PAC species. Click here for file

Additional file 3 Titel: Gene order data used for TreeREx and CREx analysis. Descriptions: Gene order data for 13 unique gene orders observed in species belonging to the Pezizomycotina and used for TreeREx and CREx analysis. Click here for file

Additional file 4 Titel: CREx and TreeREx analysis. Description: In the first two sections an informal introduction to crex and treerex is given. For a detailed introduction we refer to Bernt et al. (2007, 2008). Sections 3 to 6 include a detailed description of the gene order analyses performed completing the results included in the paper. Click here for file

Additional file 5 Titel: Polymorphism screening in PAC. Description: Amount and distribution of polymorphisms found at four mt loci for five species of the *Phialocephala fortinii* s.l. – *Acephala applanata* species complex (PAC). The four loci included approx. 7,800 bp representing 17.8% of the mt genome. The position of protein coding regions and rDNAs are indicated. Thick bars represent the fragment chosen for testing mt loci for species diagnosis in PAC (8 species, 32 strains). Click here for file

Additional file 6 Titel: List of primers used to sequence the mt genome of *Phialocephala subalpina.* Description: List of primers used to amplify and sequence the complete mt genome of *Phialocephala subalpina.* Click here for file

Additional file 7 Titel: Presence/absence of tRNA genes in species belonging to the Pezizomycotina included in crex and treerex analysis. Click here for file

Additional file 8 Titel: Primers used for phylogenetic analyis in the *Phialocephala fortinii* s.l. – *Acephala applanata* species complex (PAC). Description: Primers used to amplify three mtDNA loci in eight species belonging to PAC. Click here for file
