# Supplementary material for: Mitochondrial genome evolution in species belonging to the Phialocephala fortinii s.l. - Acephala applanata species complex
Source: BMC Genomics. 2012 May 4;13:166. doi: 10.1186/1471-2164-13-166 (PMC3434094; doi:10.1186/1471-2164-13-166)
Supplement: Additional file 2 — Titel: Protein aligenemts for putative ORFs. Description: Protein alignments of two putative ORFs (ORF_02, ORF_03) for eight PAC species. [file 1471-2164-13-166-S2.doc]

ORF_02

1_124_1 1 MNIYIKLKTNMIKALANFTLAKFCGAIFTITTLAIIKYLISGDFHLEYCDFWNNVGIGLL
2_120_3 1 MNIYIKLKTNMIKALANFTLAKFCGAIFTITTLAIIKYLISGDFHLEYCDFLNNVGIGLL
3_117_2 1 MNIYIKLKTNMIKALANFTLAKFCGAIFTITTLAIIKYLISGDFHLEYCDFWNNVGIGLL
4_123_4 1 MNIYIKLKTNMIKALANFTLAKFCGAIFTITTLAIIKYLISGDFHLEYSDFLNNVGIGLL
5_134_3 1 MNIYIKLKTNMIKALANFTLAKFCGAIFTITTLAIIKYLISGDFHLEYSDFWNNVGIGLL
6_70_1 1 MNIYIKLKTNMVKALANFTLAKFCGAIFTITTLAIIKYLISGDFHLEYSDFWNNVGIGLL
7_6_7v 1 MNIYIKLKTNMVKALANFTLAKFCGAIFTITTLAIIKYLISGDFHLEYSDFWNNVGIGLL
T1_50_2 1 MNIYIKLKTNMIKALANFTLAKFCGAIFTITTLAIIKYLISGDFHLEYSDFWNNVGIGLL


1_124_1 61 GWTINTGLIAWLTEYLGIKGINFNLNQFLFGFETMKAGETTKVGETSSTLEDGKPKLYNA
2_120_3 61 GWTINTGLIAWLTEYLGIKGINFNLNQFLFGFETMKAGETTKVGETSSALEDGKPKLYNA
3_117_2 61 GWTINTGLIAWLTEYLGIKGINFNLNQFLFGFETMKAGETTKVGETSSTLEDGKPKLYNA
4_123_4 61 GWTINTGLIAWLTEYLGIKGINFNLNQFIFGFETMKVGETTKLGETSSKVEDGKPRLYNA
5_134_3 61 GWTINTGLIAWLTEYLGIKGINFNLNQFLFGFETMKVGETTKVGETSSKVEDGKPKLYNA
6_70_1 61 GWTINTGLIAWLTEYLGIKGINFNLNQFLFGFETMKVGETTKVGETSYKVEDGKPKLYNA
7_6_7v 61 GWTINTGLIAWLTEYLGIKGINFNLNQFLFGFETMKVGETTKVGETSYKVEDGKPKLYNA
T1_50_2 61 GWTINTGLIAWLTEYLGIKGINFNLNQFIFGLETMKVGETTKVGETPSILEEGKPKLYNA


1_124_1 121 MDSGEESTSGKKSGSGKKGLNRNRDVRVHPYPRNGRRAVRSWVFDDESENGSENGSDNGS
2_120_3 121 MDSGEESTSGKKSGSGKKGLNRNRDVRVHPYPRNGRRAVRSWVFDDESENGS----DNGS
3_117_2 121 MDSGEESTSGKKSGSGKKGLNRNRDVRVHPYPRNGRRAVRSWVFDDESENGS----DNGS
4_123_4 121 MDSGEESTSGKKSGSGKKGLNRNRDVLVHPYPRNGRRAVRSWVFDDESENGSENGSDNGS
5_134_3 121 MDSVEESTSGKKSGSGKKGLNRNRDVRVHPYPRNGRRAVRSWVFDDESENGS----DNGS
6_70_1 121 MDSGEESTSGKKSGSGKKGLNRNRDVRVHPYPRNGRRAVRSWVFDDESENGSENGSDSGG
7_6_7v 121 MDSGEESTSGKKSGSGKKGLNRNRDVRVHPYPRNGRRAVRSWVFDDESENGSENGSDNGG
T1_50_2 121 MDSGEESTSGKKSGSGKKGLNRNRDVRVHPYPRNGRRAVRSWVFDDESENGSENGSNNGS


1_124_1 181 DNGSGSDTEMEGGPSNRNKNKKLAPLTSQGATQELSLDKGNANSVLPTDSLDKGKGIETA
2_120_3 177 DNGSGSDTEMEGGPSNRNKNKKLAPLTSQGATQELSLDKGNANSVLPTDSLDKGKGIETA
3_117_2 177 DNGSGSDTEMEGGPSNRNKNKKLAPLTSQGATQELSLDKGNANSVLPTDSLDKGKGIETA
4_123_4 181 DNGSGSDTEMEGGPSNRNKNKKLASLTSQGATQELSLDKGNANSVLPTDPLDKGKGIETA
5_134_3 177 DNGSGSDTEMEGGPSNRNKNKKLAPLTSQGATQELSLDKGNANSVLPTDSLDKGKGIETA
6_70_1 181 DNGSGSDTEMEGGPSNRNKNKKLASLTSQGATQELSLDKGNANSVLPTDPLDKGKGIETA
7_6_7v 181 DNGSGSDTEMEGGPSNRNKNKKLASLTSLGATQELSLDKGNANSVLPTDPLDKGKGIETA
T1_50_2 181 DNGSGSDTEMEGGPSNRNKKKKLASLTSQGATQELSLDKGNANSVLPPDPLDKGKGIETA


1_124_1 241 NASSESPISIWTRVFPGLDPTTVFFPQRTNPGPGFAVPGSEVPIQDEICQHIDYNGHILS
2_120_3 237 NASSESPISIWTRVFPGLDPTIVFFPQRTNPGPGFAVPGSEVPIQDEICQHIDYNGHILS
3_117_2 237 NASSESPISIWTRVFPGLDPTTVFFPQRTNPGPGFAVPGSEVPIQDEICQHIDYNGHILS
4_123_4 241 NASSESPISIWTRVFPGLDPTTVFFPQRTNPGPGFAVPGGEVPIQDEICQHIDYNGHILS
5_134_3 237 NASSESPISIWTRVFPGLDPTTVFFPQRTNPGPGFAVPGSEVPIQDEICQHIDYNGHILS
6_70_1 241 NASSESPISIWTRVFPGLDPTTIFFPQRTNPGPGFAVPGGEVPIQDEICQHIDYNGHILS
7_6_7v 241 NASSESPISIWTRVFPGLDPTTVFFPQRTNPGPGFAVPGGEVPIQDEICQHIDYNGHILS
T1_50_2 241 NASPESPISIWTRVIPGLDPTTVFFPQRTNPGPGFAVPGGEVPIQDEICQHIDYNGHILS


1_124_1 301 QFKNMDLETAVQQRDRYHLCVQIMSGKIAFAQEALGKVPTIPTTEYEFKLRNQISRDLDG
2_120_3 297 QFRNMDLETAVQQRDRYHLCVQIMSGKIAFAQEALGKVPTIPTTEYEFKLRNQISRDLDG
3_117_2 297 QFKNMDLETAVQQRDRYHLCVQIMSGKIAFAQEALGKVPTIPTTEYEFKLRNQISRDLDG
4_123_4 301 QFKNMDLETAVQQRDRYHLCVQIMSGKIAFAQEALGKVPTIPTTEYEFKLRNQISRDLDG
5_134_3 297 QFKNMDLETAVQQRDRYHLCIQIMSGKIAFAQEALGKVPTIPTTEYEFKLRNQISRDLDG
6_70_1 301 QFKNMDLETAVQQRDRYHLCVQIMSGKIAFAQEALGKVPTIPTTEYEFKLRNQISRDLDG
7_6_7v 301 QFKNMDLETAVQQRDRYHLCVQIMSGKIAFAQEALGKVPTIPTTEYEFKLRNQISRDLDG
T1_50_2 301 QFKNMDLETAIQQRDRYHLCVQIMSGKIAFAQEALGKVPTIPTTEHEFKLRNQISRDLDG


1_124_1 361 LNRVKVRSEARATLLNSRILFIEAQIKNNNNNN-*
2_120_3 357 LNRVKVRSEARATLLNSRILFIEAQIKNNNNNN-*
3_117_2 357 LNRVKVRSEARATLLNSRILFIEAQIKNNNNNN-*
4_123_4 361 LNRVKVRSEARATLLNSRILFIEAQIKNNNNNN-*
5_134_3 357 LNRVKVRSEARATLLNSRILFIEAQIKNNNNNN-*
6_70_1 361 LNRVKVRSEARATLLNSRILFIEAQIKNNNNNN-*
7_6_7v 361 LNRVKVRSEARATLLNSRILFIEAQIKNNNNNN-*
T1_50_2 361 LNRVKVRSEARATLLNSRILFIEAQIKNNNNNNN*

ORF_03

1_124_1 1 MNRGDWVRRYFTEQQNLRNLGIEQRLITNIMRVIARQPQTPFITQTYCRLTALNGQKIQQ
2_120_3 1 MNRGDWVRRYLTEQQNLRNLGIEQRLITNIMRVIAR*PQTPFITQTYCRLTALNGQKIQQ
3_117_2 1 MNRGDWVRRYLTEQQNLRNLGIEQRLITNIMRVIARQPQTPFITQTYCRLTALNGQKIQQ
4_123_4 1 MNRGDWVRRYFTEQANLRNLGLEQRLITNIMKVIARQPQTPFITQTCCRLTALNGQKIQQ
5_134_3 1 KNRGNWVRRYFTEQQNLRNLGIEQRLITNIMRVIARQPQTPFITQTYCRLTALNGQKIQQ
6_70_1 1 MNRGDWVRRYLTEQVNLRNLGIEQRLITNIMNVIARQPQTPFIAQTYCRLTALNGQKIQQ
7_6_7v 1 MNRGDWVRRYLTEQANLRNLGIEQRLITNIMKVIARQPQTPFIIQTYCRLTALNG*KIQQ


1_124_1 61 MVRSHRRMFIARLLIVGYRDNNNPNSPDNNLTWGDNTNNLNNNTNNSSNNNFNFNIIITL
2_120_3 60 MVRSHRRMFIARLLIVGYRDNNNPNSPDNNLTWGDNTNNLNNNTNNSSNNNFNFNIIITL
3_117_2 61 MVRSHRRMFIARLLIVGYRDNNNPNSPDNNLTWGDNTNNLNNNTNNSSNNNFNFNIIITL
4_123_4 61 MVRSNRRMYIAPLLIEGYRDNNNPNSPDNNLSWGDDTNNLNNNTNNSSNNNFNFNISITL
5_134_3 61 MVRSHRRMFIARLLIVGYRDNNNPNSPDNNLT-CDNTNNLNNNTNNSSNNNFNFNITITL
6_70_1 61 MVRSNRRMYIARLLIEGYRDNNNPNSPDNNLSWGDDTNNLNYNTNNSSNNNFNFNIIITL
7_6_7v 60 MVRSNGRMYIARLLIEGYRDNNNPNSPDNNLSWGDGTNNLNNNTNNSNNNNFNFNIIITL


1_124_1 121 SLLLLGILVITSFVILALYQVEYLYV*
2_120_3 120 SLFLLGVLVITSFVILVLYQVEYFYV*
3_117_2 121 SLLLLGVLVITSFVILALYQVEYLYV*
4_123_4 121 SLLL*GVLVITSFVILALYQVEYLYG*
5_134_3 120 SLLLLGVLVITSFVILALYQVEYLYV*
6_70_1 121 SLLLLGILVITSFVILALYQVEYLYG*
7_6_7v 120 SLLLLGVLVITSFVILALYQVEYLYG*
