# Supplementary material for: Mitochondrial genome evolution in species belonging to the Phialocephala fortinii s.l. - Acephala applanata species complex
Source: BMC Genomics. 2012 May 4;13:166. doi: 10.1186/1471-2164-13-166 (PMC3434094; doi:10.1186/1471-2164-13-166)
Supplement: Additional file 4 — Titel: CREx and TreeREx analysis. Description: In the first two sections an informal introduction to crex and treerex is given. For a detailed introduction we refer to Bernt et al. (2007, 2008). Sections 3 to 6 include a detailed description of the gene order analyses performed completing the results included in the paper. [file 1471-2164-13-166-S4.pdf]

Detailed Description of Gene Order Analysis

In the first two sections an informal introduction to `CREx` and `TreeREx` is given. For a detailed introduction we refer to Bernt et al. (2007, 2008). Sections 3 to 6 contain the details of the gene order analyses as presented in the paper.

1 CREx

`CREx` is a heuristic for computing rearrangement scenarios for pairs of gene orders that have i) the same set of genes and for which ii) each gene occurs exactly once per gene order. It considers four types of rearrangement operations, *i.e.,* inversions, transpositions, inverse transpositions, and tandem duplication random loss events. `CREx` is based on a simple formal definition for conserved gene clusters known as common intervals (Heber and Stoye, 2001). A common interval is a set of genes that appears consecutively in both gene orders. The `CREx` method is based on the observation that the different types of rearrangement operations create specific patterns in the relative order of the common intervals in two given gene orders. Thus, the basic idea of `CREx` is to search for these patterns in the common intervals of two permutations and to return the scenario consisting of the corresponding rearrangement operations. A noteworthy consequence is that `CREx` rearrangement scenarios preserve all the common intervals of the input gene orders, *i.e.,* all the intermediate gene orders also have these common intervals.

2 TreeREx

Given a phylogenetic tree, `TreeREx` maps pairwise rearrangement scenarios computed by `CREx` to the branches of the tree. Thereby it reconstructs also the ancestral gene orders at each node of the tree except for the root node.

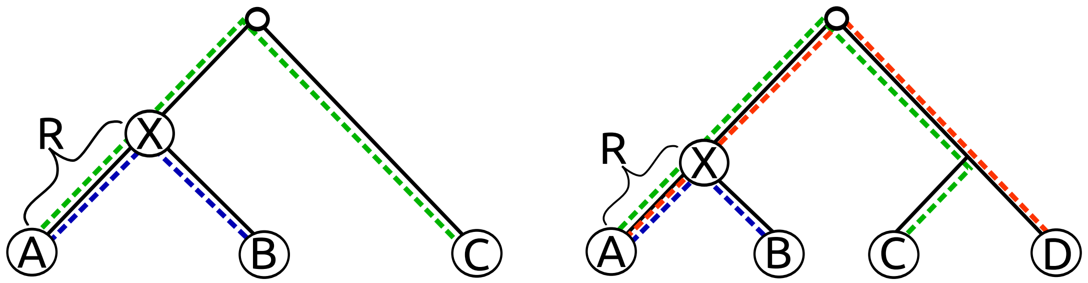

Figure 1: Illustration of the idea of `TreeREx`; the used pairwise `CREx` scenarios are indicated by dashed lines.

The idea of `TreeREx` is described with the help of the phylogeny shown in Figure 1 (left). The rearrangement scenario R on the branch from X to A is determined by computing the pairwise rearrangement scenarios from the other two nodes towards A, *i.e.,* from B to A and from C to A. The reconstructed rearrangement scenario R includes exactly those rearrangements, which are included in both scenarios. Similarly, the pairwise `CREx` scenarios from A and C to B are used for determining the scenario from X to B.

In order to verify that the reconstructed rearrangement scenarios are correct, the rearrangements predicted

on the branch XA are (de)applied starting from X and those mapped to branch XB are (de)applied starting from B. If the resulting gene orders are identical it can be assumed that this is the ancestral gene order at node X and the reconstructed rearrangements are correct. In this case the reconstruction for node X is called *consistent*.

In the case of a tree with four leaves the same idea can easily be applied (see Figure 1 (right)). In order to reconstruct R, the `CREx` scenarios from all other leaves to A are computed, *i.e.,* from B, C, and D, and the common rearrangements are included in R.

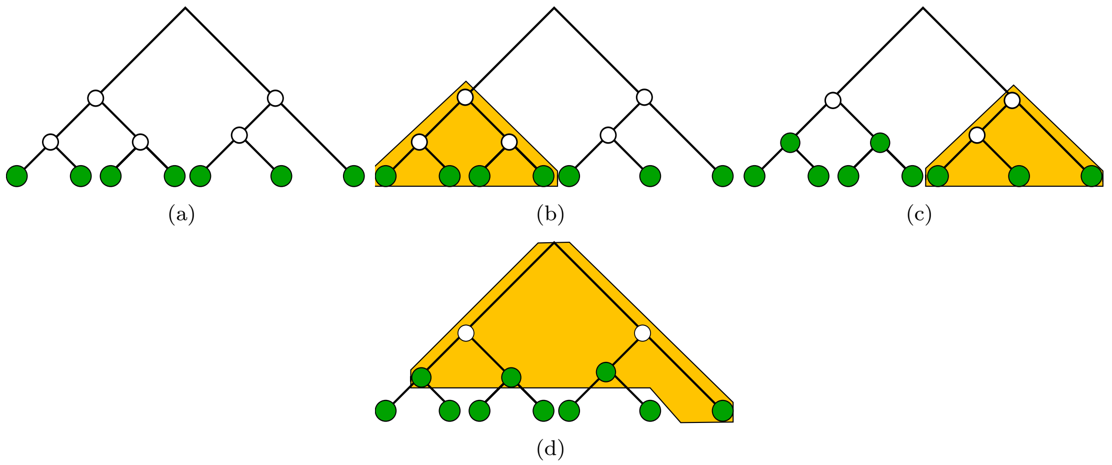

Figure 2: Illustration of the bottom up strategy implemented in `TreeREx` from (a) to (d); Green nodes have an assigned gene order; Subtrees with three or four leaves that are considered are highlighted in orange.

This procedure can fail if one or more of the `CREx` scenarios are wrong. Similarly, a wrong phylogenetic tree can be the source of such a failure. Therefore, `TreeREx` tries to find a solution by ignoring one or more of the pairwise rearrangement scenarios. For instance in the four leaf case a reconstruction for R is made from the scenarios B to A and C to A (ignoring D to A); furthermore a potentially different reconstruction is made using the scenarios B to A and D to A (ignoring C to A). If one of both is consistent then it is considered and if both are correct a parsimonious solution is taken. This is called 1-consistent. If also this procedure fails a *fallback* mode is employed. In the three leaf tree, `TreeREx` chooses the ancestral gene order from all possible intermediate states from A to B (and B to A) minimizing the number of rearrangements to C. In the four leaf case, a pair of intermediate states from A to B (and B to A) and C to D (and D to C) with minimum distance is chosen.

Since the quality of `CREx` reconstructions tends to decrease for distant genomes the above idea is not generalized to more than four leaves. Instead a given phylogenetic tree is analyzed in a bottom-up manner by iteratively considering triples and quadruples of gene orders (see Figure 2).

3 `TreeREx` Analysis of the Data Set

The data set was analyzed using all gene orders excluding *Podospora anserina*, *Mycosphaerella graminicola*, and *Phaeosphaeria nodorum*. The gene set included in the analysis was composed of 14 OXPHOS proteins

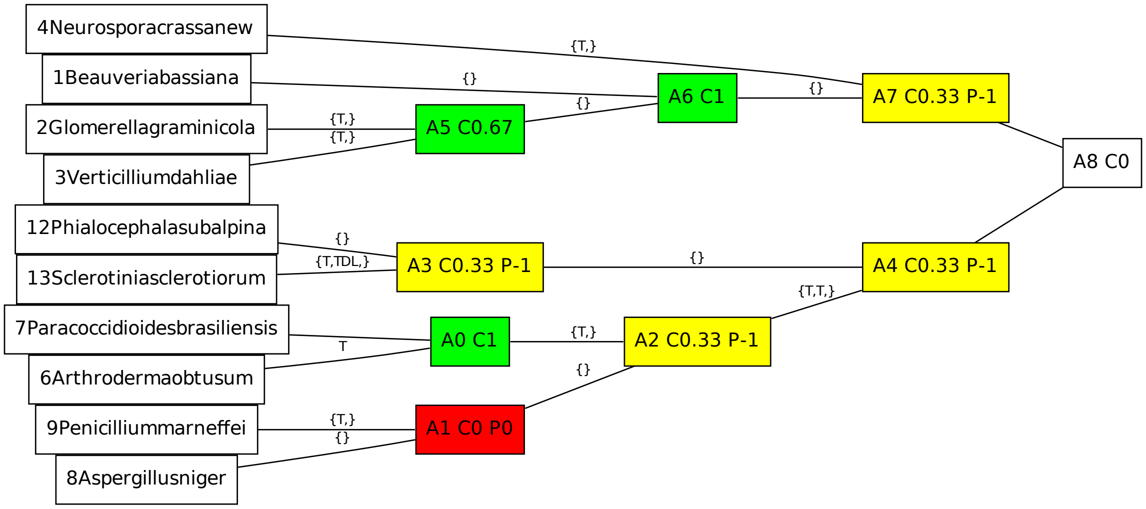

Figure 3: Original output of CREx and TreeREx analysis. The rearrangements on the branches are given as T for a transposition and TDL for tandem-duplication-random-loss events; green nodes marks consistent reconstructed nodes; yellow indicates 1-consistent reconstructed nodes; and red nodes are reconstructed with the fallback method. The value following the "P" in the node label shows how much better the chosen solution is in comparison with other possible solution(s); Rearrangements on the branches to the root node of the phylogenetic tree are not shown.

(atp6, atp8, atp9, cox1-3, cob, nad1-6, nad4L), Rps3 and the two rRNA genes (rnl and rns), *i.e.,* 17 genes. The tree used as backbone in the TreeREx analysis was derived from the phylogenetic analysis of the protein data of 12 OXPHOS genes (see paper for more details). The result of `TreeREx` is shown in Figure 3. All nodes, except the node labeled A1, were reconstructed consistently or 1-consistent. In all cases where a 1-consistent solution was reconstructed it was locally more parsimonious then alternative solutions. The node A1 was reconstructed with the fallback method and there is one more solution with the same number of rearrangements and one more non-parsimonious reconstruction. This is locally with respect to the gene orders of the subtree, *i.e.,* A0, the gene order of *P. marneffei* (group 8), and the gene order of *A. niger* (group 9).

In order to increase the confidence in the reconstruction of node A1, we have manually inspected all combinations of different ancestral states for nodes A1, A2, and A3 with `CREx` (see Figure 4). Parsimonious combinations are listed in Table 1.

There are four parsimonious solutions for this subtree, *i.e.,* solutions needing six rearrangements. Note that, in these reconstructions A1 is always reconstructed as A1a, *i.e.,* the gene order of *Aspergillus niger.* The first line – which is the solution predicted by `TreeREx`– is not only parsimonious within the subtree, but also parsimonious with respect to the rearrangements to the node A7 (see Table 2). The second solution (A3b, A2b, A1a) has one more rearrangement for A3, but one less for A2. That is, within the subtree its as parsimonious as the solution given by `TreeREx`. But to connect to A7 two more additional transpositions are necessary. The last two of these reconstructions (A3g,A2a,A1a and A3g,A2b,A1a) imply one more transposition to A7 due to the choice of A3g compared to `TreeREx`' solution. Furthermore both include one tdrl instead of a transposition which can be regarded as a more complicated hypothesis. Thus the solution returned by `TreeREx` is the most parsimonious among the analyzed.

4 `CREx` Scenarios for the Dothideomycetes

The Dothideomycetes do not posses the same set of genes as the other species. Actually, *atp8* and *atp9* are missing in *P. nodorum* and *M. graminicola* do not include *Rps3.* Therefore `TreeREx` cannot be applied. But, the `CREx` scenarios can be computed with the 15 common genes.

From *P. nodorum* to *M. graminicola* `CREx` predicts three inversions and two tdrls. In the other direction even one tdrl more. From the predicted ancestral state to *M. graminicola* two inverse transpositions, two inversions, one transpositions, two tdrls; and from A to *P. nodorum* one inverse transposition, 2 inversions, and 3 tdrls.

This is a very large number of rearrangements. In particular with respect to the tdrls these pairwise comparisons are quite "saturated". This is, because the maximum number of tdrls necessary to reach any gene order starting from an arbitrary other gene order is $\lceil \log_2 n \rceil$ (Chaudhuri et al., 2006), where n is the number of genes, *i.e.,* four tdrls for this data set. Presenting a rearrangement scenario is further complicated by the fact that there are often many shortest tdrl scenarios for a given pair of gene orders (Bernt et al., 2011).

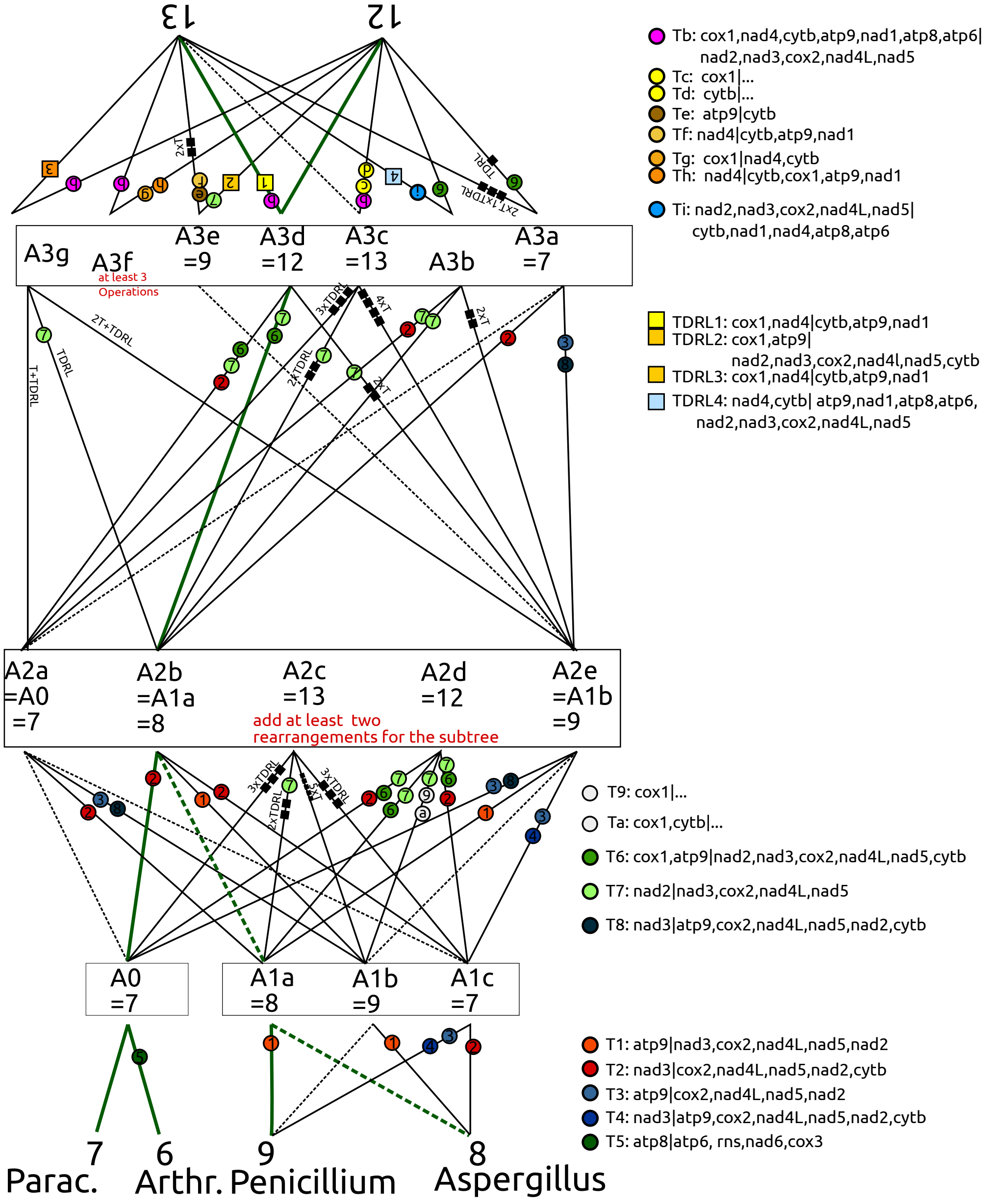

Figure 4: Analysis of non-local influences of the choice of the ancestral state for the node A1 (A2 and A3). The ancestral nodes of the tree are shown as rectangles including the ancestral states proposed by `TreeREx` (*e.g.,* a-g). Transpositions are shown as colored circles T1,...,Th and tdrls as colored square TDRL1-3; X|Y specifies the actual rearrangements. For a transposition the genes in X are transposed with the genes in Y. For a tdrl the genes in $X \cup Y$ are duplicated, the genes in X are kept in the first and those in Y in the second copy; scenarios that can not be part of a more parsimonious combination are just indicated and combinations of A3f and A2b/A2c are not included for the same reason. The combination of ancestral states chosen by `TreeREx` is highlighted in green; dashed lines indicate that no rearrangements take place.

A3 to 12 & 13			A2 to A3			A2 to A0 and A1 to 8 & 9			
A3	#	R	A2	#	R	A1	#	R	$\sum$#
d	2	T tdrl	b	2	2T	a	2	2T	6
b	3	2T tdrl	b	1	T	a	2	2T	6
g	2	T tdrl	a	2	T tdrl	a	2	2T	6
g	2	T tdrl	b	2	T tdrl	a	2	2T	6
d	2	T tdrl	a	3	3T	a	2	2T	7
d	2	T tdrl	b	2	2T	b	3	3T	7
b	3	2T tdrl	a	2	2T	a	2	2T	7
b	3	2T tdrl	b	1	T	b	3	3T	7
g	2	T tdrl	a	2	T tdrl	b	3	3T	7
g	2	T tdrl	a	2	T tdrl	c	3	3T	7
g	2	T tdrl	b	2	T tdrl	b	3	3T	7
g	2	T tdrl	e	3	T tdrl	b	3	3T	8
d	2	T tdrl	a	3	3T	b	3	3T	8
d	2	T tdrl	a	3	3T	c	3	3T	8
d	2	T tdrl	e	3	3T	b	3	3T	8
c	3	3T	a	3	3 tdrl	a	2	2T	8
c	3	3T	b	3	T tdrl	a	2	2T	8
b	3	T tdrl	a	2	2T	b	3	3T	8
b	3	T tdrl	a	2	2T	c	3	3T	8
b	3	T tdrl	e	2	2T	b	3	3T	8
a	5	T tdrl	b	1	T	a	2	2T	8
e	6	5T tdrl	b	1	4T	a	2	2T	9
e	6	5T tdrl	e	0	2T	b	3	3T	9
g	2	T tdrl	b	2	T tdrl	c	5	4T	9
g	2	T tdrl	e	3	T tdrl	a	4	4T	9
d	2	T tdrl	b	2	2T	c	5	4T	9
d	2	T tdrl	e	3	3T	a	4	4T	9
c	3	3T	a	3	3 tdrl	b	3	3T	9
c	3	3T	a	3	3 tdrl	c	3	3T	9
c	3	3T	b	3	T tdrl	b	3	3T	9
b	3	2T tdrl	b	1	T	c	5	4T	9
b	3	2T tdrl	e	2	2T	a	4	4T	9
a	5	3T tdrl	a	2	2T	a	2	2T	9
a	5	3T tdrl	b	1	T	b	3	3T	9

Table 1: All combinations of the possible ancestral states proposed by `TreeREx` (see Figure 4) that need less than 10 rearrangements; The first line gives the reconstruction of `TreeREx`; The three parts of the of the table show from left to right: i) the rearrangements from node A3 to 12 and 13, ii) the rearrangements from node A2 to A3 ii) the rearrangements from note A2 to A0, A1, and from A1 to 8 and 9. The ancestral state of A0 is not varied and T5 not counted. The columns give the index of the variants used for the nodes A1, A2, A3, the number of rearrangements (#), and the types of rearrangements; the last column which is used for sorting gives the sum of the rearrangements.

A2a A3a	A2b	A2c A3c	A2d A3d	A2e A3e	A3f	A3g
T iT I tdrl	4T	2T 2tdrl	2T	4T	T 2tdrl	3T

Table 2: Rearrangements computed by `CREx` for the possible ancestral states from A2 and A3 to A7; equal gene orders are given in the same column.

5 `CREx` Scenario for *P. anserina*

Also *P. anserina* lacks the *atp9* gene, which rules out the use of `TreeREx`. By excluding the *atp9* gene `CREx` can be applied for a comparison with the gene orders of *B. bassiana* (group 1 in Figure 4 of the paper) and *N. crassa* (group 4 in Figure 4 of the paper). The pairwise comparison of the three gene orders is complicated by the fact that the common intervals of the three pairs are partially in conflict, *i.e.,* rearrangements that are allowed in one comparison may be forbidden in another. Furthermore, in the comparisons configurations of gene clusters of the form ABC in the one gene order and CBA in the other can be found. For instance, $A = \{cob, cox1\}$; $B = \{nad4L, nad5\}$; $C = \{cox2\}$ and $A = \{rns, cox3, nad6\}$, $B = \{atp6\}$, $C = \{cob, cox1, nad4L, nad5, cox2, nad4, atp8, nad1\}$ in the comparison of *B. bassiana* and *P. anserina*. Because there is no transposition or tdrl scenario which preserves the common intervals, *i.e.,* the clusters $\{A, B\}$ and $\{B, C\}$, `CREx` can only explain this by a combination of inverse transposition and inversions. But inverse transpositions may be considered unlikely in a data set completely lacking inverted genes and it seems to be preferable to use transpositions or tdrls. Hence, some of the common intervals have to be ignored in order to yield a common rearrangement scenario for the three gene orders.

For each of the pairwise comparison we manually identified the rearrangements that `CREx` reconstructed based on its pattern for a transpositions. These rearrangements have been organized in a graph displaying putative rearrangement scenarios for the three gene orders (see Figure 5).

For the comparison of *B. bassiana* with *N. crassa* a singe transposition is predicted (Tδ, *i.e.,* the transposition 1 in Figure 4C in the paper). Transpositions Tζ and Tι are found as transposition patterns in the comparison of *N. crassa* with *P. anserina*, but they conflict with the strong intervals of the pair *P. anserina* and *B. bassiana*. The argument favoring these is as follows, any other transposition affecting these gene clusters is also in conflict with the common intervals of *P. anserina* and *B. bassiana*, but in addition also with the common intervals of *P. anserina* and *N. crassa*. Therefore Tζ and Tι may be considered less invasive. In both comparisons with *P. anserina* transposition Tκ is found and may therefore be considered as confident. From the gene order X resulting from applying Tζ, Tι, and Tκ to the gene order of *P. anserina* two transpositions to *B. bassiana* are predicted, *i.e.,* Tα and Tβ. We considered only these rearrangements since they are also compliant with respect to the gene order X and that of *N. crassa* but any other rearrangements compliant with the common intervals of X and *N. crassa* is not compliant with the common intervals of X and *B. bassiana*. Since the two possible intermediate gene orders of the transformation from X to *B. bassiana* via Tα and Tβ need only one more transposition (Tγ and Tϵ, respectively) also these should be considered. There are several scenarios needing 6 transpositions: From X to *P. anserina* via Tζ, ι, κ plus

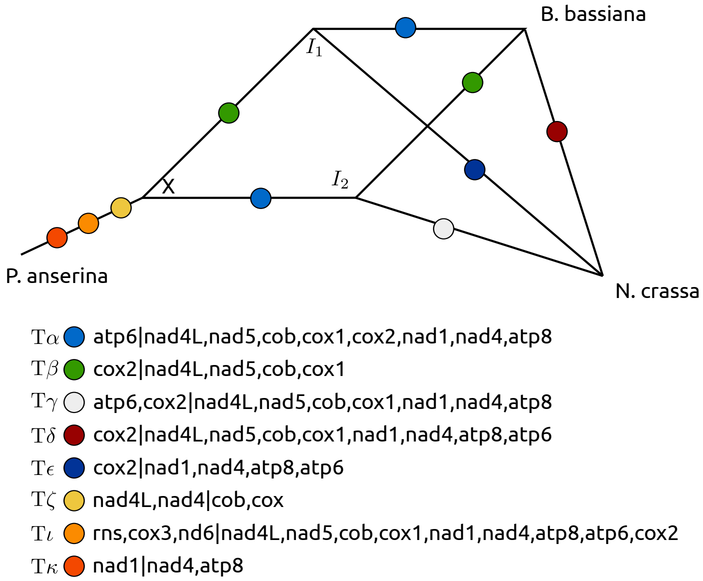

Figure 5: Putative rearrangement scenarios for *B. bassiana*, *N. crassa*, and *N. anserina*

1. *B. bassiana* to *N. crassa* via Tδ and from *B. bassiana* to X via Tα, β, *i.e.,* the gene order of *B. bassiana* ancestral for *P. anserina* and *N. crassa*;
2. *B. bassiana* to *N. crassa* via Tδ and from *N. crassa* to X via Tγ, α or Tϵ, β, *i.e.,* the gene order of *N. crassa* is ancestral;
3. *B. bassiana* to I_1 via Tα and from there via Tβ and Tϵ to X and *N. crassa*, respectively, *i.e.,* I_1 is ancestral;
4. *B. bassiana* to I_2 via Tβ and from there via Tα and Tγ to X and *N. crassa*, respectively, *i.e.,* I_2 is ancestral.

The differences in these rearrangement scenarios concern the transposition of the gene *cox2*. In *B. bassiana* the gene is in the front part of the gene order and in *P. anserina* and *N. crassa* the gene is in the back of the gene order (with respect to the chosen linearisation). The rearrangements that are involved in the movement of *cox2* are T$\beta, \gamma, \delta, \epsilon$. The common property of the scenarios is that *cox2* is moved twice.
Within the transpositions $\alpha, \beta, \gamma, \delta, \epsilon$ only Tδ is detected by `CREx` in one of the pairwise comparisons of the three gene orders. Furthermore the comparison of the gene orders of *B. bassiana* and *N. crassa* needs the fewest rearrangements and a small number of rearrangements can be considered more confident (Bernt and Middendorf, 2011). Therefore, the first of the above variants is depicted in the paper.

6 Intermediate Position of gene order of Leotiomycetes

A detailed analysis of `CREx` scenarios for the ancestral gene orders for the Sordariomycetes, Leotiomycetes, and Eurotiomycetes predicted by `TreeREx` has been carried out. We have computed all pairwise rearrangement scenarios for the putative ancestral gene orders G1, A, and G8 with `CREx` (see Figure 4 in the paper).

Since the transposition of *cox3* and *nad6* appears from G8 as well as from A to G1 we can assume that this transposition happened towards G1. Therefore, we consider gene order G1' which is G1 with applied transposition.

For G1' to G8, G1' to A, and A to G8 all possible intermediate gene orders have been enumerated, *i.e.,* the gene orders which will be passed when applying the rearrangements in any order permutation (all rearrangements do commute). These gene orders have been organized in a graph using the gene orders as vertices. Two vertices are connected by an edge if a single transposition separates the corresponding gene orders (see Figure 6). From this network we select a smallest subset of the edges connecting the three input gene orders (in technical terms a "minimum spanning tree"). Except for the order of the two rearrangements separating A and G there is a unique solution, which is indicated as red line in the Figure. This is a parsimonious explanation for the input gene orders - which has the putative ancestral gene orders of Leotiomycetes in an intermediate position. This suggests that A is indeed the ancestral state on the sister clade of the Sordariomycetes as shown in Figure 4 of the paper.

However, the rearrangements on the red edges can be mapped easily on all three possible phylogenies connecting the three gene orders G1, G8, and A.

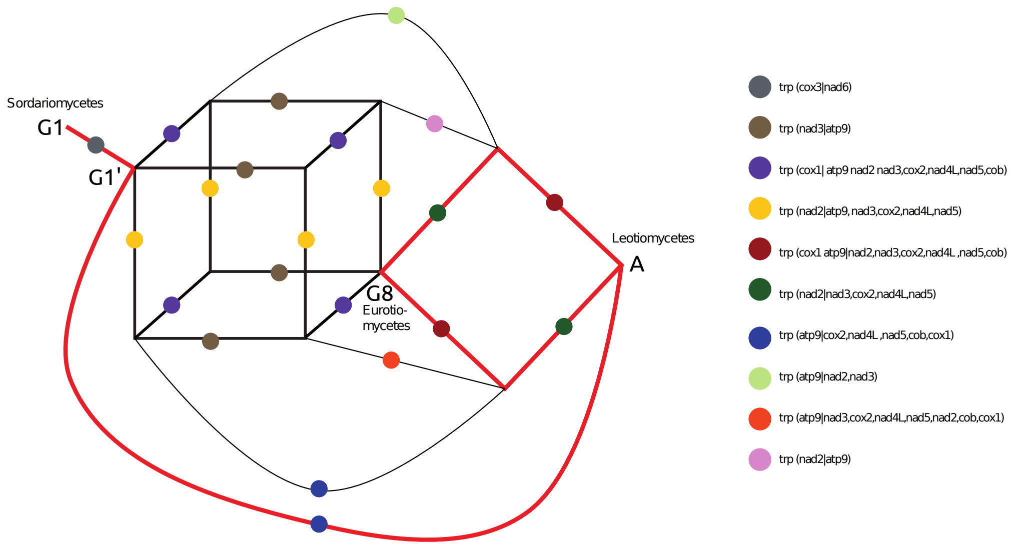

Figure 6: Detailed analysis of parsimonious scenarios of gene order evolution among the hypothesized ancestral gene orders found in the Sordariomycetes, Leotiomycetes and Eurotiomycetes. Parsimonious pairwise transposition scenarios reconstructed with `CREx` for the gene orders and possible intermediate gene orders are presented. The predicted transpositions are indicated on the left showing the transposed gene clusters separated by "|". The rearrangements highlighted in red describe a parsimonious solution for the putative ancestral gene orders.

References

Bernt, M., Chen, K.-Y., Chen, M.-C., Chu, A.-C., Merkle, D., Wang, H.-L., Chao, K.-M., and Middendorf, M. (2011). Finding all sorting tandem duplication random loss operations. *Journal of Discrete Algorithms*,

9(1).

Bernt, M., Merkle, D., and Middendorf, M. (2008). An algorithm for inferring mitogenome rearrangements in a phylogenetic tree. In *Comparative Genomics, International Workshop, RECOMB-CG 2008, Proceedings*, volume 5267 of *Lecture Notes in Bioinformatics*, pages 143–157. Springer.

Bernt, M., Merkle, D., Ramsch, K., Fritzsch, G., Perseke, M., Bernhard, D., Schlegel, M., Stadler, P. F., and Middendorf, M. (2007). CREx: inferring genomic rearrangements based on common intervals. *Bioinformatics*, 23(21):2957–2958.

Bernt, M. and Middendorf, M. (2011). A method for computing an inventory of metazoan mitochondrial gene order rearrangements. *BMC Bioinformatics*, 12(Suppl 9):S6.

Chaudhuri, K., Chen, K., Mihaescu, R., and Rao, S. (2006). On the tandem duplication-random loss model of genome rearrangement. In *Proceedings of the Seventeenth Annual ACM-SIAM Symposium on Discrete Algorithms, SODA 2006*, pages 564–570, New York, NY, USA. ACM.

Heber, S. and Stoye, J. (2001). Finding all common intervals of k permutations. In *Combinatorial Pattern Matching, 12th Annual Symposium, CPM 2001, Proceedings*, volume 2089 of *Lecture Notes in Computer Science*, pages 207–218. Springer.
